# Supplementary figures and images for: Nationally endorsed learning objectives to improve course design in introductory biology
Source: PLoS One. 2024 Aug 15;19(8):e0308545. doi: 10.1371/journal.pone.0308545 (PMC11326583; doi:10.1371/journal.pone.0308545)

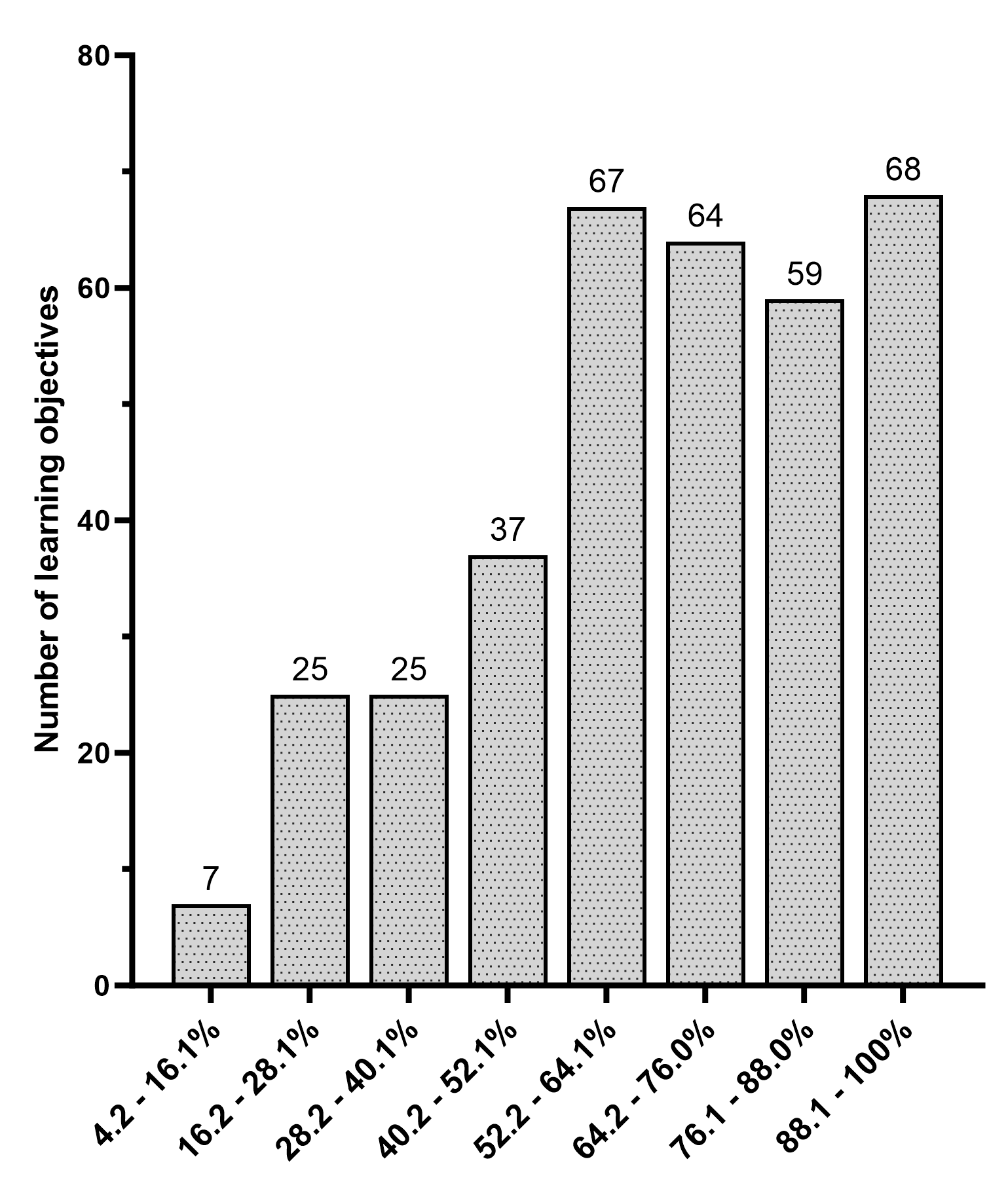

Supplement: S1 Fig — NOTE: This was reviewed/endorsed by Groups 2 and 3. (TIF) [file pone.0308545.s002.tif]

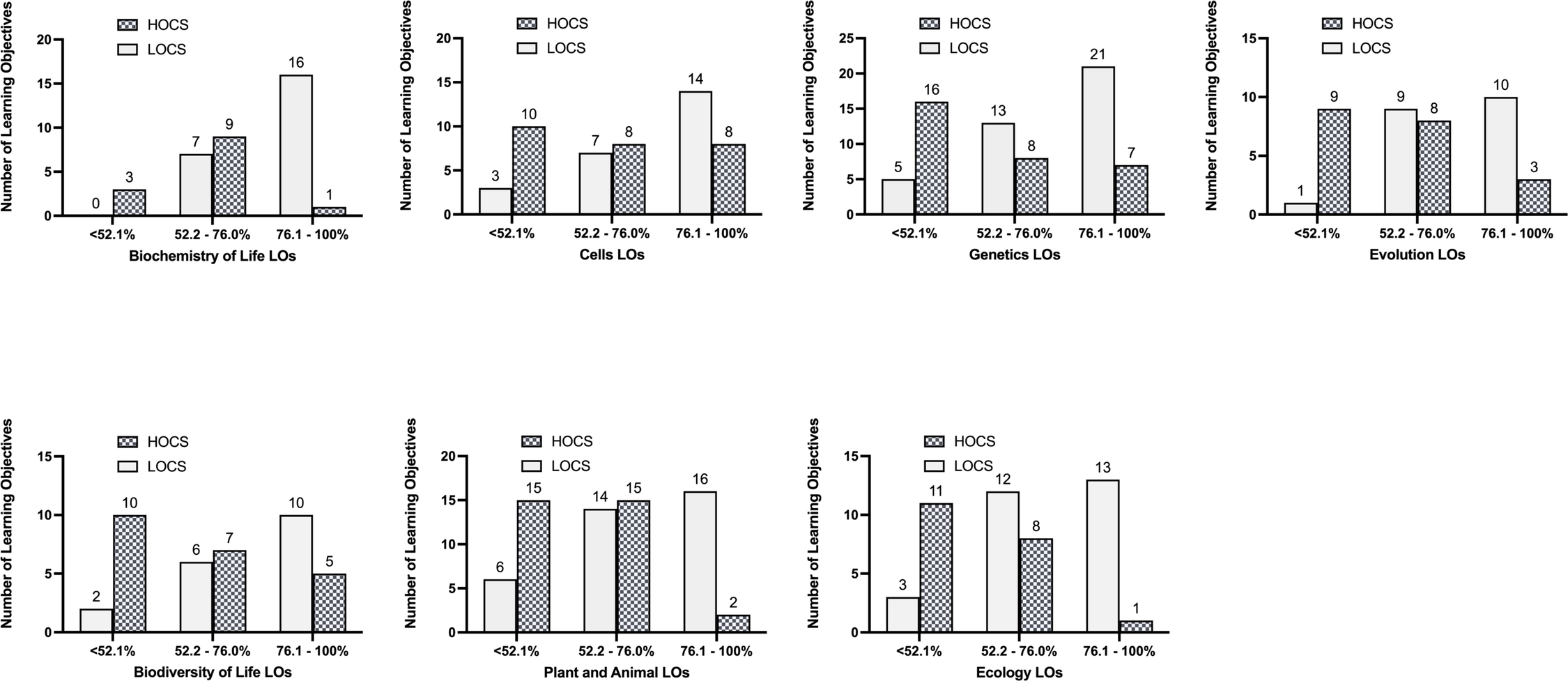

Supplement: S2 Fig — (TIF) [file pone.0308545.s003.tif]

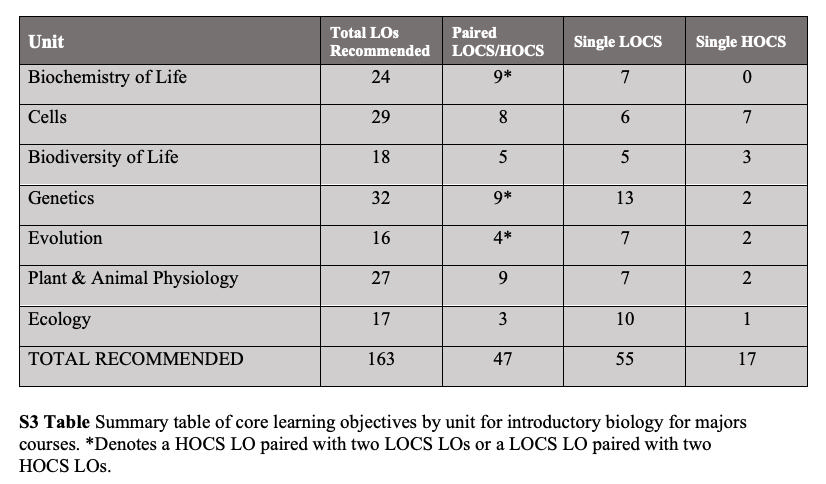

Supplement: S1 Table — *Denotes a HOCS LO paired with two LOCS LOs or a LOCS LO paired with two HOCS LOs. (TIF) [file pone.0308545.s004.tif]
