# Supplementary material for: Nationally endorsed learning objectives to improve course design in introductory biology
Source: PLoS One. 2024 Aug 15;19(8):e0308545. doi: 10.1371/journal.pone.0308545 (PMC11326583; doi:10.1371/journal.pone.0308545)
Supplement: S1 Text — (PDF) [file pone.0308545.s005.pdf]

Welcome

This NSF-supported project will support biology educators by providing a set of learning objectives for the Introductory Biology for Majors series.

Once these proposed learning objectives have been validated by you and other biology instructors as essential for Introductory Biology for Majors courses, we will make them available to the community as a resource to facilitate course design and improve assessment.

- You may exit and return to the survey as needed.
- The survey automatically resumes where you left off if you use the same browser and do not clear cookies.

Have you ever served as the instructor of record for the Introductory Biology for Majors series?

- ○ Yes
- ○ No

Please choose to evaluate a unit that you teach on a regular basis. You will receive a randomized subset of learning objectives from that unit.

You may take the unit survey again if you wish to evaluate additional learning objectives. You may also evaluate learning objectives from a different unit.

Thank you for your participation.

- ○ LEARNING OBJECTIVE FOR PLANT AND ANIMAL PHYSIOLOGY
- ○ LEARNING OBJECTIVES FOR GENETICS
- ○ LEARNING OBJECTIVES FOR EVOLUTION
- ○ LEARNING OBJECTIVE FOR ECOLOGY
- ○ LEARNING OBJECTIVES FOR BIODIVERSITY OF LIFE
- ○ LEARNING OBJECTIVES FOR CELLS

○ LEARNING OBJECTIVES FOR BIOCHEMISTRY OF LIFE

Powered by Qualtrics

Respondent Characteristics

UNIT: BIOCHEMISTRY OF LIFE

We ask that you complete the following demographic questions so that we can determine if we are gathering feedback from a representative population. We will not link your responses with any individual identifying information when sharing the results of this survey.

Please skip to the bottom of this section if you are returning to the survey and have already provided us with this information.

What is the name of your current institution?

____

Which of the following best describes your institution type?

- ○ Associate's Degree-Granting
- ○ Bachelor's Degree-Granting
- ○ Master's Degree-Granting
- ○ Doctoral Degree-Granting
- ○ Other (Please Specify)

Which of the following best describes your current position?

- ○ Postdoc
- ○ Lecturer, Instructor, or Teaching Faculty
- ○ Assistant, Associate, or Full Professor
- ○ Staff

- Other (Please Specify)

In your current position, what is your <u>primary</u> responsibility?

- Teaching
- Research
- Teaching and Research Equally
- Other (Please Describe Briefly)

What is or was the focus of your graduate training? (Please select all that apply.)

- Molecular/Cellular/Developmental Biology
- Anatomy/Physiology
- Ecology/Evolutionary Biology
- Discipline-Based Education Research
- Other (Please Specify)

In an average academic year when you are teaching, which levels do you teach? (Please select all that apply.)

- Non-Majors Lower-Level (100 - 200 Level)
- Majors Lower-Level (100 - 200 Level)
- Upper-Level (300 - 400 Level)
- Graduate-Level (500+ Level)

What is the primary focus of the majority of biology courses that you have taught? (Please select all that apply.)

- ☐ Biochemistry
- ☐ Cell Biology
- ☐ Genetics
- ☐ Evolution
- ☐ Biodiversity of Life
- ☐ Plant and Animal Physiology
- ☐ Ecology
- ☐ Other (Please Specify)

In a typical academic term *when you are teaching in an Introductory Biology for Majors series*, what is the focus of the course you teach? (Please select all that apply.)

- ☐ Biochemistry
- ☐ Cell Biology
- ☐ Genetics
- ☐ Evolution
- ☐ Biodiversity of Life
- ☐ Plant and Animal Physiology
- ☐ Ecology
- ☐ Other (Please Specify)

To what extent do you communicate learning objectives to your students in your introductory biology course? (Please select all that apply.)

- [ ] Every class session
- [ ] Weekly
- [ ] Unit overview
- [ ] Course overview
- [ ] Syllabus
- [ ] Other (Please Briefly Describe)

Biochemistry of Life: Classes of Biological Molecules, Water, Nucleic Acids

This block of ***fifteen*** learning objectives is part of the **BIOCHEMISTRY OF LIFE** unit, under the topics:

- **COMPARING THE MAJOR CLASSES OF BIOLOGICAL MACROMOLECULES**
- **WATER**
- **NUCLEIC ACIDS**

Please note that the learning objectives you are evaluating are a subset of all of the learning objectives proposed for the unit.

Please indicate whether the following learning objectives are **essential** or **non-essential** for the course you teach.

TOPIC: COMPARING THE MAJOR CLASSES OF BIOLOGICAL MOLECULES

	ESSENTIAL	NON-ESSENTIAL
1. Compare the monomer subunit, bond responsible for polymerization, and important biological function(s) observed in proteins, nucleic acids, and carbohydrates.	○	○
2. Compare the primary, secondary, and tertiary structures of proteins, RNA, and DNA.	○	○
3. Analyze how the structure of biological molecules impacts their function, including explaining the connections among the following three statements: 1) amino acids are much more diverse in structure and chemical properties than nucleotides, 2) in terms of diversity in shape and chemical properties, proteins > RNA > DNA, and 3) in terms of diversity in function, proteins > RNA > DNA.	○	○
4. Define the term amphipathic and give examples of proteins and lipids that are and are not amphipathic.	○	○

Please share any feedback you have about the content or wording of the above learning objectives.

Please indicate whether the following learning objectives are **essential** or **non-essential** for the course you teach.

TOPIC: WATER

	ESSENTIAL	NON-ESSENTIAL
1. Draw the structure of several water molecules that are interacting and indicate 1) the electron distributions in each covalent bond, 2) the partial charges on each atom, and 3) each hydrogen bond.	○	○
2. On a structural model of a molecule you haven't seen before, draw the electron distributions on covalent bonds between O, C, N, and H atoms based on their relative electronegativities. Then 1) label any partial or full charges on the molecule's atoms, and 2) predict whether the molecule will be soluble in water. Explain your reasoning using a drawing.	○	○
3. Compare hydrogen bonds and covalent bonds in terms of the mechanisms and strength of attraction between the atoms involved.	○	○
4. Explain the relationship between hydrogen bonding and phenomena such as sweating, moderate coastal climates, and the oceans' response to global warming.	○	○
5. Define the terms acid, base, and pH. Sketch the pH scale and note where on the scale you find strong acids, strong bases, and water.	○	○

Please share any feedback you have about the content or wording of the above learning objectives.

Please indicate whether the following learning objectives are **essential** or **non-essential** for the course you teach.

TOPIC: NUCLEIC ACIDS

	ESSENTIAL	NON-ESSENTIAL
1. Draw the general structure of a nucleotide using a pentagon for the sugar, a box for the nitrogenous base, and a circle for the phosphate group.	○	○
2. Given a structural model of a molecule, identify whether it is a ribonucleotide or deoxyribonucleotide, and then 1) label the 3' and 5' carbon, 2) add a second nucleotide as if polymerization had occurred, and 3) label the 5' and 3' ends of the 2-base-long strand.	○	○
3. Make or label drawings that describe the primary and secondary structure of DNA, noting the location of phosphodiester bonds and hydrogen bonds and explaining their role in stabilizing both levels of structure.	○	○
4. Define complementary base pairing, and explain its connection to the observation that DNA strands are antiparallel.	○	○
5. Use the pairing rules to 1) explain the observation that in DNA, %A = %T and %G = %C, 2) predict the sequence of a complementary strand of DNA when given one strand, and 3) calculate the percentage of each base in a DNA molecule when given the percentage of one base.	○	○
6. Compare 1) the primary and secondary structures of DNA and RNA, 2) their relative stability, 3) their sizes and shapes, and 4) their functions.	○	○

Please share any feedback you have about the content or wording of the above learning objectives.

Biochemistry of Life: Carbohydrates, Proteins, Lipids

This block of ***twenty-one*** learning objectives is part of the **BIOCHEMISTRY OF LIFE** unit, under the topics:

- **CARBOHYDRATES**
- **PROTEINS**
- **LIPIDS**

Please note that the learning objectives you are evaluating are a subset of all of the learning objectives proposed for the unit.

Please indicate whether the following learning objectives are **essential** or **non-essential** for the course you teach.

TOPIC: CARBOHYDRATES

	ESSENTIAL	NON-ESSENTIAL
1. Draw the general structure of a monosaccharide, disaccharide, and polysaccharide and give an example of each.	○	○
2. In terms of their chemical structure, explain the fundamental differences between carbohydrates that function in energy storage and use versus those that provide structural support.	○	○

	ESSENTIAL	NON-ESSENTIAL
3. Predict the biological function of a polysaccharide you've never seen before based on 1) the structure of the monosaccharides present, and 2) the type of linkage that creates the polymer. (The type of linkage will be labeled.)	○	○

Please share any feedback you have about the content or wording of the above learning objectives.

Please indicate whether the following learning objectives are **essential** or **non-essential** for the course you teach.

TOPIC: PROTEINS

	ESSENTIAL	NON-ESSENTIAL
1. Label the four components of an amino acid and explain the role of each in terms of how the molecule functions in a protein.	○	○
2. Predict whether the R-group on an amino acid that you haven't seen before will 1) interact with water, and 2) act as an acid (proton donor) or base (proton acceptor).	○	○
3. Describe each of the four levels of protein structure and explain how each influences the protein's final size, shape, and chemical properties.	○	○

	ESSENTIAL	NON-ESSENTIAL
4. Label elements of primary, secondary, tertiary, and quaternary structure on a model of a protein that you haven't seen before.	○	○
5. Compare which bonds are responsible for producing a protein's 1) primary structure, 2) secondary structure (alpha-helices and beta-pleated sheets), and 3) tertiary structure.	○	○
6. Given a model that shows the key bonds responsible for a protein's secondary or tertiary structure, predict 1) whether a change in a specific amino acid will change the protein's structure, and 2) which aspects of structure will be altered first in response to heating, changes in pH, or other conditions.	○	○
7. Describe at least three functions that proteins serve in cells.	○	○

Please share any feedback you have about the content or wording of the above learning objectives.

Please indicate whether the following learning objectives are **essential** or **non-essential** for the course you teach.

TOPIC: LIPIDS

	ESSENTIAL	NON-ESSENTIAL
1. Use drawings, models, or other representations to compare the structures of fats, phospholipids, and steroids.	○	○
2. Given a structural model of a lipid you've never seen before, 1) identify it as a fat, phospholipid, or steroid, 2) determine if it is saturated or unsaturated, and 3) predict its function in the cell.	○	○
3. Label the hydrophilic head and hydrophobic tails on a drawing of a phospholipid, then make drawings that include water molecules to explain how phospholipids spontaneously form bilayers in water.	○	○
4. Given several models of membranes, predict how differences in phospholipid composition and cholesterol content will affect their relative fluidity and permeability, and explain your reasoning.	○	○
5. Draw a cell membrane and label integral and peripheral proteins, carbohydrate components, and lipid components.	○	○
6. Compare the processes of diffusion, osmosis, and facilitated diffusion, and provide biological examples that illustrate each process.	○	○
7. Explain why ions and polar molecules do not move across plasma membranes efficiently without a transport protein.	○	○
8. Given several ions and molecules, predict the relative rates at which they will cross a plasma membrane in the absence of membrane proteins. Explain your reasoning.	○	○

	ESSENTIAL	NON-ESSENTIAL
9. Define passive and active transport and explain the role of channels, carriers, and pumps in transport.	○	○
10. Create a drawing, model, or other representation to illustrate the existence of an electrochemical gradient across a plasma membrane.	○	○
11. Given a diagram of an electrochemical gradient, predict 1) which direction a specified ion or molecule will cross a plasma membrane, and 2) whether a channel, carrier, or pump would be involved.	○	○

Please share any feedback you have about the content or wording of the above learning objectives.

Demographic Questions

Do you have Biology Education Research experience?

Yes	No
○	○

Other (Please Specify) ○

To what extent have the Vision and Change **Core Concepts** changed your Introductory Biology course design since the report was issued by the AAAS in 2011?

A Great Deal Some

Very Little None

To what extent have the Vision and Change **Core Competencies** changed your Introductory Biology for Majors course design since the report was issued by the AAAS in 2011?

A Great Deal Some

Very Little None

I most closely identify as

Woman Man

Non-binary/Trans

Other (Please Specify)

Prefer not to say

I most closely identify as (Please select all that apply)

American Indian or Alaska Native

☐

Asian or Asian American

☐

Black or African American

☐

Hispanic, Latinx, or Spanish origin

☐

Native Hawaiian or Pacific Islander

☐

White

☐

Multiracial

☐

Other (Please Specify)

☐

Prefer not to say

☐

Thank you for reviewing a block of assigned learning objectives for an Introductory Biology course for Majors. If you are interested in providing feedback on additional blocks of learning objectives, please select **REVIEW ADDITIONAL LOs.**

REVIEW ADDITIONAL LOs

○

FINISH SURVEY

○

If you have any questions about the project or this survey, please do not hesitate to contact me, Dr. Kelly Hennessey (hennek@uw.edu), or Dr. Scott Freeman (srf991@uw.edu).

Powered by Qualtrics

Respondent Characteristics

UNIT: CELLS

We ask that you complete the following demographic questions so that we can determine if we are gathering feedback from a representative population. We will not link your responses with any individual identifying information when sharing the results of this survey.

Please skip to the bottom of this section if you are returning to the survey and have already provided us with this information.

What is the name of your current institution?

Which of the following best describes your institution type?

- Associate's Degree-Granting
- Bachelor's Degree-Granting
- Master's Degree-Granting
- Doctoral Degree-Granting
- Other (Please Specify)

Which of the following best describes your current position?

- Postdoc
- Lecturer, instructor, or teaching faculty
- Assistant, Associate, or Full Professor
- Staff

Other (Please Specify)

In your current position, what is your primary responsibility?

- Teaching
- Research
- Teaching and Research Equally
- Other (Please Describe Briefly)

What is or was the focus of your graduate training? (Please select all that apply.)

- Molecular/Cellular/Developmental Biology
- Anatomy/Physiology
- Ecology/Evolutionary Biology
- Discipline-Based Education Research
- Other (Please Specify)

In an average academic year when you are teaching, which levels do you teach? (Please select all that apply.)

- Non-Majors Lower-Level (100 - 200 Level)
- Majors Lower-Level (100 - 200 Level)
- Upper-Level (300 - 400 Level)
- Graduate-Level (500+ Level)

What is the primary focus of the majority of biology courses that you have taught? (Please select all that apply.)

- [ ] Biochemistry
- [ ] Cell Biology
- [ ] Genetics
- [ ] Evolution
- [ ] Biodiversity of Life
- [ ] Plant and Animal Physiology
- [ ] Ecology
- [ ] Other (Please Specify)

In a typical academic term *when you are teaching in an Introductory Biology for Majors series*, what is the focus of the course you teach? (Please select all that apply.)

- [ ] Biochemistry
- [ ] Cell Biology
- [ ] Genetics
- [ ] Evolution
- [ ] Biodiversity of Life
- [ ] Plant and Animal Physiology
- [ ] Ecology
- [ ] Other (Please Specify)

To what extent do you communicate learning objectives to your students in your introductory biology course? (Please select all that apply.)

- [ ] Every class session
- [ ] Weekly
- [ ] Unit overview
- [ ] Course overview
- [ ] Syllabus
- [ ] Other (Please Briefly Describe)

Cells: Overview; Inside the Cell

This block of ***seventeen*** learning objectives is part of the **CELLS** unit, under the topics:

- **OVERVIEW**
- **INSIDE THE CELL**

Please note that the learning objectives you are evaluating are a subset of all of the learning objectives proposed for the unit.

Please indicate whether the following learning objectives are **essential** or **non-essential** for the course you teach.

TOPIC: OVERVIEW

	ESSENTIAL	NON-ESSENTIAL
1. Given the summary reactions for photosynthesis and respiration, compare 1) the reactants and products of each process, and 2) the energy transformations that occur.	○	○

	ESSENTIAL	NON-ESSENTIAL
2. Predict how an array of events involving photosynthesis and cellular respiration impact atmospheric CO_2 levels, such as extensive tree planting programs or oxidation of newly thawed carbohydrates by soil organisms in what used to be arctic permafrost.	○	○
3. Predict whether photosynthesis and/or cellular respiration will occur in a specific plant or animal cell, based on information about the cell's structure and function.	○	○

Please share any feedback you have about the content or wording of the above learning objectives.

Please indicate whether the following learning objectives are essential or non-essential for the course you teach.

TOPIC: INSIDE THE CELL

	ESSENTIAL	NON-ESSENTIAL
1. Compare key elements of prokaryotic versus eukaryotic cell structure.	○	○

	ESSENTIAL	NON-ESSENTIAL
2. Propose hypotheses to explain 1) the adaptive significance of organelles (the advantages and disadvantages of having membrane-bound structures inside cells), and 2) why organelles are more common in eukaryotes than bacteria and archaea.	○	○
3. Compare key elements of plant versus animal cell structure.	○	○
4. Propose a hypothesis to explain the adaptive significance of major differences in the structure of plant and animal cells.	○	○
5. Predict what would happen to a cell if a particular organelle or structure was altered in a specified manner.	○	○
6. Predict 1) the function of a cell, given a drawing, micrograph, or description of its structure and organelle content, and 2) the structure of a cell, given information on its function. In each case, explain your reasoning.	○	○
7. Make a flow chart showing how proteins are processed and packaged or unpackaged as they move from ribosomes to the interior of the rough ER to Golgi to motor proteins to their destination.	○	○
8. Predict what would happen to a particular protein or overall cell function if a specified element or process in the endomembrane system were altered.	○	○

	ESSENTIAL	NON-ESSENTIAL
9. Explain the relationship between molecular "zip code" signals and receptor molecules in ensuring that proteins are delivered to their correct destination in the cell.	○	○
10. Analyze data or design an experiment on where a specific protein is synthesized, transported, and used in the cell, given a method for labeling that protein.	○	○
11. Compare the structure and function of microtubules, actin filaments (microfilaments), and intermediate filaments.	○	○
12. Create a hypothesis to explain why cytoskeletal elements are more common in eukaryotes than bacteria and archaea.	○	○
13. Make a labeled drawing illustrating how motor proteins use chemical energy to change shape and create movement in the cell.	○	○
14. Describe one or more types of evidence you would need to see to be convinced that a newly discovered protein functions as a motor in the cell.	○	○

Please share any feedback you have about the content or wording of the above learning objectives.

Cells: Cell Cycle and Mitosis; Enzymes and Energetics

This block of ***sixteen*** learning objectives is part of the **CELLS** unit, under the topics:

- **CELL CYCLE AND MITOSIS**
- **ENZYMES AND ENERGETICS**

Please note that the learning objectives you are evaluating are a subset of all of the learning objectives proposed for the unit.

Please indicate whether the following learning objectives are **essential** or **non-essential** for the course you teach.

TOPIC: CELL CYCLE AND MITOSIS

	ESSENTIAL	NON-ESSENTIAL
1. Given a micrograph or drawing of a cell you've never seen before, label the chromosomes, chromatids, sister chromatids, and homologous chromosomes, if present, and determine the haploid number and ploidy.	○	○
2. Explain why chromosome replication has to occur before mitosis, in interphase.	○	○

	ESSENTIAL	NON-ESSENTIAL
3. Given a micrograph or drawing of a cell you've never seen before that is undergoing mitosis, explain what is currently happening to the chromosomes.	○	○
4. Given a labeled drawing showing the phases of mitosis, explain what is happening to the chromosomes and how it helps ensure that each daughter cell gets a complete and identical set.	○	○
5. Analyze the causes of abnormal chromosome numbers in the daughter cells of mitosis.	○	○
6. Diagram the sequence of stages in the eukaryotic cell cycle (M, G1, S, and G2) and label the major event or events that occur in each.	○	○
7. Predict the consequences of altering a given stage (M, G1, S, and G2) in the cell cycle in terms of the cell's structure or fate.	○	○
8. Describe the importance of cell cycle checkpoints in general terms, identify the distinctive roles of the G1/S, G2/M, and metaphase checkpoints, and explain the role of activating or deactivating regulatory molecules in triggering each phase.	○	○

Please share any feedback you have about the content or wording of the above learning objectives.

Please indicate whether the following learning objectives are **essential** or **non-essential** for the course you teach.

TOPIC: ENZYMES AND ENERGETICS

	ESSENTIAL	NON-ESSENTIAL
1. Given a graph showing how free energy changes over the course of a chemical reaction, 1) label the sections representing the reactants, activation energy, and products, 2) explain why energy peaks during the transition state, and 3) determine whether the reaction is exergonic or endergonic.	○	○
2. Explain 1) why "active site" is an appropriate term, 2) the mechanisms responsible for the observation that enzymes lower activation energies, 3) why most enzymes catalyze one specific reaction, and 4) why enzymes increase reaction rates but do not make endergonic reactions exergonic.	○	○
3. Given a structural model that shows the R-groups in an active site and reaction substrates in a transition state, predict the consequences for enzyme function if one R-group is replaced with a different R-group.	○	○

	ESSENTIAL	NON-ESSENTIAL
4. Make labeled "cartoons" comparing the structure of an active site when 1) the enzyme is unregulated, 2) a competitive inhibitor is bound, and 3) an allosteric inhibitor is bound.	○	○
5. Given structural models showing different regulatory molecules bound to the same site of an enzyme, predict 1) which will bind more or less tightly, and 2) the consequences of tighter or looser binding for enzyme function.	○	○
6. Interpret graphs of reaction rate versus pH, temperature, and degree of substrate saturation for a given enzyme. Based on your analysis, predict the nature of the cell's normal environment in nature.	○	○
7. Explain 1) the general role of ATP in the cell, 2) what it means to say that two chemical reactions are coupled, and 3) why a large change in free energy level occurs when an enzyme or substrate is phosphorylated. (Recall that phosphorylation adds 3 tightly packed negative charges.)	○	○
8. Given graphs showing how free energy changes over the course of a chemical reaction, predict whether two specific reactions can be successfully coupled.	○	○

Please share any feedback you have about the content or wording of the above learning objectives.

Cells: Cellular Respiration; Photosynthesis

This block of ***seventeen*** learning objectives is part of the **CELLS** unit, under the topics:

- **CELL RESPIRATION**
- **PHOTOSYNTHESIS**

Please note that the learning objectives you are evaluating are a subset of all of the learning objectives proposed for the unit.

Please indicate whether the following learning objectives are **essential** or **non-essential** for the course you teach.

TOPIC: CELL RESPIRATION

	ESSENTIAL	NON-ESSENTIAL
1. Make a chart summarizing the inputs and outputs of glycolysis, pyruvate processing, the citric acid cycle, and oxidative phosphorylation, using NADH, $FADH_2$, Glucose, Acetyl CoA, Pyruvate, O_2, CO_2, H+ gradients, and ATP. Using the chart, explain how energy is transferred or transformed in each stage.	○	○
2. Predict the possible consequences if a step in the glucose oxidation (cellular respiration) pathway is altered.	○	○

	ESSENTIAL	NON-ESSENTIAL
3. Given the overall formula for aerobic cellular respiration, label each of the following: 1) the atoms that lose electrons and the atoms that gain electrons, and 2) the molecules that are reduced and the molecules that are oxidized.	○	○
4. Consider the reactions that impact C-C, C-H, and C-O bonds during cellular respiration and explain 1) the changes in electron position that occur, and 2) the consequences in terms of gain or loss of potential energy.	○	○
5. Explain how cells use fermentation pathways to obtain energy from glucose in the absence of oxygen.	○	○
6. Analyze the costs and benefits of pushing yourself -- during exercise or competition--until oxygen is depleted in your muscles.	○	○
7. Draw and label a "cartoon" model of electron donors, the electron transport chain, the final electron acceptor, and ATP synthase that shows the path of electrons, the direction of the electrochemical gradient established by the ETC, and the path of protons. Add notes indicating 1) the relative amount of potential energy in electrons held by NADH versus oxygen, and 2) where ADP is phosphorylated to ATP.	○	○
8. Predict the effects of altering specific parts of the electron transport chain or ATP synthase.	○	○

	ESSENTIAL	NON-ESSENTIAL
9. Draw a cartoon model of ATP synthase and explain how it functions as a "turbine that responds to a flow of protons."	○	○
10. Make a diagram illustrating in general terms how feedback mechanisms control cellular respiration, providing an example of a step that is regulated. Explain how regulation of this step maximizes efficiency.	○	○
11. Predict what might happen if a step in a feedback mechanism that controls cell respiration is disrupted.	○	○

Please share any feedback you have about the content or wording of the above learning objectives.

Please indicate whether the following learning objectives are **essential** or **non-essential** for the course you teach.

TOPIC: PHOTOSYNTHESIS

	ESSENTIAL	NON-ESSENTIAL
1. Given the overall formula for photosynthesis, label the atoms that lose electrons, the atoms that gain electrons, the molecules that are reduced, and the molecules that are oxidized. Explain the impacts of these changes on the energy available in each molecule.	○	○

	ESSENTIAL	NON-ESSENTIAL
2. Explain to an 8-year-old how the CO_2 in "weightless" air is the source of mass in a redwood tree.	○	○
3. Diagram the ultraviolet, visible, and infrared ranges of the electromagnetic spectrum, add an arrow showing the direction of increased energy, and put stars at the regions that are most active in driving photosynthesis.	○	○
4. Given data on the wavelengths of light available in different habitats, explain how photosynthesizers could be adapted to use them.	○	○
5. Make a chart summarizing the inputs and outputs of PSI, PSII, and the Calvin cycle using NADPH, Glucose, H_2O, O_2, CO_2, H+ gradients, and ATP. Using this chart, explain the energy transformations that occur and the role of rubisco.	○	○
6. Predict the possible consequences for the production of ATP and NADPH if a component or process in the photosynthesis pathway is altered.	○	○

Please share any feedback you have about the content or wording of the above learning objectives.

Demographic Questions

Do you have Biology Education Research experience?

Yes No

Other (Please Specify)

To what extent have the Vision and Change **Core Concepts** changed your Introductory Biology course design since the report was issued by the AAAS in 2011?

A Great Deal Some

Very Little None

To what extent have the Vision and Change **Core Competencies** changed your Introductory Biology course design since the report was issued by the AAAS in 2011?

A Great Deal Some

Very Little None

I most closely identify as

Woman Man

Non-binary/Trans

Other (Please Specify)

Prefer not to say

I most closely identify as

American Indian or Alaska Native

Asian or Asian American

Black or African American

Hispanic, Latinx, or Spanish origin

Native Hawaiian or Pacific Islander

White

Multiracial

Other (Please Specify)

☐

Prefer not to say

☐

Thank you for reviewing a block of assigned learning objectives for an Introductory Biology course for Majors. If you are interested in providing feedback on additional blocks of learning objectives, please select **REVIEW ADDITIONAL LOs.**

REVIEW ADDITIONAL LOs

○

FINISH SURVEY

○

If you have any questions about the project or this survey, please do not hesitate to contact me, Dr. Kelly Hennessey (hennek@uw.edu), or Dr. Scott Freeman (srf991@uw.edu).

Powered by Qualtrics

Respondent Characteristics

UNIT: GENETICS

We ask that you complete the following demographic questions so that we can determine if we are gathering feedback from a representative population. We will not link your responses with any individual identifying information when sharing the results of this survey.

Please skip to the bottom of this section if you are returning to the survey and have already provided us with this information.

What is the name of your current institution?

____

Which of the following best describes your institution type?

- ○ Associate's Degree-Granting
- ○ Bachelor's Degree-Granting
- ○ Master's Degree-Granting
- ○ Doctoral Degree-Granting
- ○ Other (Please Specify) __________

Which of the following best describes your current position?

- ○ Postdoc
- ○ Lecturer, instructor, or teaching faculty
- ○ Assistant, Associate, or Full Professor
- ○ Staff

- ○ Other (Please Specify)

In your current position, what is your primary responsibility?

- ○ Teaching
- ○ Research
- ○ Teaching and Research Equally
- ○ Other (Please Describe Briefly)

What is or was the focus of your graduate training? (Please select all that apply.)

- ☐ Molecular/Cellular/Developmental Biology
- ☐ Anatomy/Physiology
- ☐ Ecology/Evolutionary Biology
- ☐ Discipline-Based Education Research
- ☐ Other (Please Specify)

In an average academic year when you are teaching, which levels do you teach? (Please select all that apply.)

- ☐ Non-Majors Lower-Level (100 - 200 Level)
- ☐ Majors Lower-Level (100 - 200 Level)
- ☐ Upper-Level (300 - 400 Level)
- ☐ Graduate-Level (500+ Level)

What is the primary focus of the majority of biology courses that you have taught? (Please select all that apply.)

- ☐ Biochemistry
- ☐ Cell Biology
- ☐ Genetics
- ☐ Evolution
- ☐ Biodiversity of Life
- ☐ Plant and Animal Physiology
- ☐ Ecology
- ☐ Other

In a typical academic term, *when you are teaching in an Introductory Biology for Majors series*, what is the focus of the course you teach? (Please select all that apply.)

- ☐ Biochemistry
- ☐ Cell Biology
- ☐ Genetics
- ☐ Evolution
- ☐ Biodiversity of Life
- ☐ Plant and Animal Physiology
- ☐ Other (Please Specify)

To what extent do you communicate learning objectives to your students in your introductory biology course? (Please select all that apply.)

- ☐ Every class session

- ☐ Weekly
- ☐ Unit overview
- ☐ Course overview
- ☐ Syllabus
- ☐ Other (Please Briefly Describe)

Genetics - Meiosis; Mutations/Cancer; Patterns of Inheritance

This block of ***twenty-six*** learning objectives is part of the **GENETICS** unit, under the topics:

- **MEIOSIS**
- **MUTATIONS/CANCER**
- **PATTERNS OF INHERITANCE**

Please note that the learning objectives you are evaluating are a subset of all of the learning objectives proposed for the unit.

Please indicate whether the following learning objectives are **essential** or **non-essential** for the course you teach.

TOPIC: MEIOSIS

	ESSENTIAL	NON-ESSENTIAL
1. Explain the differences between somatic cells and germ cells. Describe the outcomes of cell division between these two categories of cells.	○	○

	ESSENTIAL	NON-ESSENTIAL
2. Differentiate between the genetic information held on two homologous chromosomes, two nonhomologous chromosomes, two sister chromatids, and two non-sister chromatids.	○	○
3. Given a micrograph or drawing of a cell you've never seen before, label chromosomes, chromatids, sister chromatids, and homologous chromosomes, if present, and determine the haploid number and ploidy of the cell.	○	○
4. Given labeled drawings showing the phases of meiosis I and II, add labels for the alleles AaBb and explain how the events of each phase affect the genotype of the daughter cells.	○	○
5. Given a micrograph or drawing of a cell you've never seen before that is undergoing meiosis, explain what is currently happening to the chromosomes.	○	○
6. Explain why the segregation of homologous chromosomes in meiosis I leads to a reduction in ploidy.	○	○
7. Given a specific error in meiosis, predict the haploid genotypes that result and discuss the consequences for offspring.	○	○
8. Draw a crossing-over event in a cell with n = 2 and the genotype AaBb. List the genotypes of the haploid cells that result with and without crossing over between genes A and B and explain why crossing over can contribute to offspring with new phenotypes.	○	○

	ESSENTIAL	NON-ESSENTIAL
9. Given any pair of homologous chromosomes, predict the haploid genotypes and associated phenotypes that result from one or more crossing over events that occur at specified points.	○	○
10. Explain why no two haploid cells that result from meiosis are alike in terms of genotype and why this is important in terms of offspring fitness.	○	○
11. Compare sexual and asexual reproduction in terms of the mechanisms involved and fitness advantages and disadvantages.	○	○
12. Analyze data or design an experiment on the hypothesis that sexual reproduction (meiosis) evolved as an adaptation against rapidly evolving viruses and disease-causing organisms.	○	○

Please share any feedback you have about the content or wording of the above learning objectives.

Please indicate whether the following learning objectives are **essential** or **non-essential** for the course you teach.

TOPIC: MUTATIONS/CANCER

	ESSENTIAL	NON-ESSENTIAL
1. Rank the following mutations in terms of greatest to least impact on the structure and function of genes and gene products: missense (change amino acids), nonsense (change to "stop"), frameshift (change reading frame), and silent (no change in the product). Explain your reasoning.	○	○
2. Rank aneuploidy, chromosome deletions, and chromosome translocations in terms of their impact on the structure and function of genes and gene products. Explain your reasoning.	○	○
3. Analyze data or design an experiment to explain why chromosomes with deletions, translocations, or abnormal numbers are found in most cancerous cells.	○	○
4. Defend the statement "mutation is the ultimate source of genetic variation," and explain why mutation is random with respect to its impact on an individual's fitness.	○	○
5. Explain why cancer is 1) associated with mutations that regulate the cell cycle, and 2) more common in older than younger people.	○	○

Please share any feedback you have about the content or wording of the above learning objectives.

Please indicate whether the following learning objectives are **essential** or **non-essential** for the course you teach.

TOPIC: PATTERNS OF INHERITANCE

	ESSENTIAL	NON-ESSENTIAL
1. Label which elements in a Punnett square represent the genotypes of egg, sperm, and offspring. Explain how you can determine the frequency of each egg and sperm genotype and how you can use this information to calculate the frequencies of offspring genotypes and phenotypes.	○	○
2. Given any pair parental genotypes and information on the alleles present, use a Punnett square to complete a genetic cross. Identify the genotypes and phenotypes of offspring and calculate their predicted frequencies. Note that the genes involved may be autosomal, X-linked, linked, or unlinked and that the alleles involved may be dominant, recessive, or co-dominant.	○	○
3. Given information on parental and offspring phenotypes, determine whether the alleles involved are 1) dominant, recessive, or codominant, 2) autosomal or X-linked, and 3) linked or unlinked.	○	○
4. Analyze data or design an experiment to test the hypothesis that a trait in a model organism, such as fur or flower color, is influenced by 1) gene-by-gene interactions or 2) gene-by-environment interactions.	○	○
5. Define polygenic inheritance and explain why it produces traits with a continuous variation.	○	○
6. Analyze data or design an experiment on trait variation to determine if a polygenic inheritance is occurring.	○	○

	ESSENTIAL	NON-ESSENTIAL
7. Using a drawing that shows the phases of meiosis, label the events that explain Mendel's principles of segregation and independent assortment. Add drawings to show how independent assortment can generate genetic variation in offspring. In each case, explain your reasoning.	○	○
8. On a pedigree, label 1) males and females, 2) affected and unaffected individuals, and 3) generations.	○	○
9. Based on the data in a pedigree, predict 1) whether the trait in question is autosomal or sex-linked and 2) which alleles are dominant and recessive.	○	○

Please share any feedback you have about the content or wording of the above learning objectives.

Genetics: DNA Replication; Information Processing

This block of ***nineteen*** learning objectives is part of the **GENETICS** unit, under the topics:

- **DNA REPLICATION**
- **INFORMATION PROCESSING**

Please note that the learning objectives you are evaluating are a subset of all of the learning objectives proposed for the unit.

Please indicate whether the following learning objectives are **essential** or **non-essential** for the course you teach.

TOPIC: DNA REPLICATION

	ESSENTIAL	NON-ESSENTIAL
1. Describe the function of major components of the replisome: helicase, topoisomerase, DNA polymerase, DNA ligase, and primase.	○	○
2. Use a drawing that you create to explain the statement: "A newly synthesized DNA strand is half old and half new."	○	○
3. Given a diagram of a DNA molecule during replication, label the following: the origin of replication, directions of replication, replication fork, the leading strand, and lagging strands and their polarities, and the replisome.	○	○
4. Using everyday objects like twine or fabric strips, create a model of DNA replication and use it to explain 1) why lagging strand synthesis is an appropriate name, and 2) why Okazaki fragments occur.	○	○
5. Using a drawing, explain 1) the problem that arises during lagging strand synthesis at the end of a chromosome, and 2) the role of telomerase in solving the problem.	○	○
6. Explain how DNA damage and/or mismatches are detected and repaired.	○	○
7. Given a specific type of DNA mutation or damage, predict which DNA repair pathway would respond, then predict the consequences of successful and unsuccessful repair.	○	○

Please share any feedback you have about the content or wording of the above learning objectives.

Please indicate whether the following learning objectives are **essential** or **non-essential** for the course you teach.

TOPIC: INFORMATION PROCESSING

	ESSENTIAL	NON-ESSENTIAL
1. Compare the structure, chemical composition, location, and function of DNA with RNA.	○	○
2. Make a flow chart summarizing the flow of information in cells from gene to protein. Label arrows connecting mRNA, DNA, and proteins, and explain what each arrow represents.	○	○
3. Add elements to your central dogma model that represent "exceptions" such as 1) production of rRNA, tRNA, and "other RNAs", 2) DNA replication, and 3) the action of an enzyme called reverse transcriptase, which catalyzes the synthesis of DNA from an RNA template.	○	○
4. Explain how the genetic code relates transcription to translation and why it is considered redundant.	○	○
5. Make a chart that compares the structure and function of the RNAs involved in information processing in cells: tRNA, rRNA, mRNA, siRNA/miRNA, and snRNPs.	○	○

	ESSENTIAL	NON-ESSENTIAL
6. Evaluate the claim that the information in this chart supports the hypothesis that in the earliest forms of life, genes and most key cellular "machines" consisted of RNA, and that DNA evolved later.	○	○
7. On diagrams of transcription initiation and transcription elongation, label the template and coding strands, initiation complex, promoter site, RNA polymerase, ribonucleotides, the direction of RNA polymerase movement, and direction of RNA synthesis.	○	○
8. Use a copy of the genetic code to predict the sequence of the amino acids produced from a given mRNA or double-stranded DNA fragment. Identify the start and stop codon.	○	○
9. Given a specific change in a DNA coding strand or a specific error in transcription or translation, predict the consequences for the gene product.	○	○
10. On diagrams of translation initiation, translation elongation, and translation termination, label the small and large ribosomal subunits, mRNA, tRNA, rRNA, reading frame, start codon, stop codon, release factor, and tRNA binding sites (E, A, and P). Circle and label the locations where codon-anticodon recognition and peptide bond formation occur.	○	○
11. Consider DNA replication, transcription, and translation, and for each process explain at least one key difference in how they occur in bacterial versus eukaryotic cells.	○	○

	ESSENTIAL	NON-ESSENTIAL
12. Given information on where a given antibiotic binds to molecules essential for transcription or translation, explain 1) why the antibiotic disrupts the process, and 2) why the disruption affects only bacterial cells--not eukaryotic cells.	○	○

Please share any feedback you have about the content or wording of the above learning objectives.

Genetics: Control of Gene Expression; Biotechnology

This block of ***twenty-five*** learning objectives is part of the **GENETICS** unit, under the topics:

- **CONTROL OF GENE EXPRESSION**
- **BIOTECHNOLOGY**

Please note that the learning objectives you are evaluating are a subset of all of the learning objectives proposed for the unit.

Please indicate whether the following learning objectives are **essential** or **non-essential** for the course you teach.

TOPIC: CONTROL OF GENE EXPRESSION

	ESSENTIAL	NON-ESSENTIAL
1. Explain how negative and positive control over transcription regulates the activity of a given gene or operon.	○	○

	ESSENTIAL	NON-ESSENTIAL
2. Analyze data or information in a case study to determine if negative or positive control of transcription is occurring.	○	○
3. Create a model of an operon that includes three genes. Label the genes, promotor, repressor binding site, and activator binding site. Add sigma, RNA polymerase, and an activator and/or a repressor to show the operon during activation or repression.	○	○
4. Make a diagram showing the condensed and decondensed states of chromatin, with labels indicating histones and nucleosomes. Using your model, explain the role of DNA methylation and histone modification in changing the state of chromatin.	○	○
5. Analyze data or design an experiment to test the hypothesis that a specific combination of histone modifications is required to keep a particular stretch of chromatin in an uncondensed state.	○	○
6. Analyze data or design an experiment on the hypothesis that changes in chromatin state can be passed on to offspring.	○	○
7. Given a model of a eukaryotic gene, label the promoter, transcription start site, start codon, stop codon, exons, introns, poly-A signal sequence, silencers, and enhancers. Explain the role of each in initiating or inhibiting transcription.	○	○
8. Given information on mutations that affect the promoter, splice sites in introns, silencers, and/or enhancers, predict their impact on the level or nature of gene expression.	○	○

	ESSENTIAL	NON-ESSENTIAL
9. Given a diagram of a pre-mRNA as it is being processed, label exons, introns, the 5' cap, and poly(A) tail, and snRNPs or the spliceosome, and explain the role of each.	○	○
10. Given information on a mutation that affects a splice site in introns, the placement or composition of the 5' cap, or the length of a poly(a) tail, predict its impact on the structure or amount of protein product.	○	○
11. Explain how alternative splicing yields more than one kind of mature, processed RNA--and thus more than one protein product--from a single gene.	○	○
12. Given a primary transcript that contains introns and exons, predict the mRNAs that could result from alternative splicing, and the consequences for the resulting gene products.	○	○
13. Explain the role of phosphorylation, dephosphorylation, and ubiquitination in regulating the activity and lifespan of proteins.	○	○
14. Using what you have learned about control of gene expression, explain 1) how bacterial cells are able to change the gene products they produce in response to changes in environmental conditions, and 2) why different cell types in the same plant or animal produce different gene products.	○	○

Please share any feedback you have about the content or wording of the above learning objectives.

Please indicate whether the following learning objectives are **essential** or **non-essential** for the course you teach.

TOPIC: BIOTECHNOLOGY

	ESSENTIAL	NON-ESSENTIAL
1. Given a list of components required for a PCR, explain the function of each and its connection to the components required for DNA replication in the cell.	○	○
2. Make a flow chart showing the steps needed to complete two cycles in a PCR experiment. Use your model to explain how many additional copies of the target sequence would be amplified in future cycles.	○	○
3. Design an appropriate PCR primer pair to amplify a given region of DNA.	○	○
4. Explain how voltage (a separation of charge) is used to separate different sizes of DNA or protein molecules in gel electrophoresis.	○	○
5. Given an image of a gel, predict the relative size of protein or DNA samples and interpret the presence or absence of a known protein or region of DNA (such as a PCR product).	○	○

	ESSENTIAL	NON-ESSENTIAL
6. Using a drawing, explain how traditional Sanger sequencing works. Explain the similarities and differences of this approach to one or more methods currently being used to sequence entire genomes.	○	○
7. Analyze bioinformatics data or design a bioinformatics study on an array of topics, such as 1) creating a hypothesis for the function of a newly discovered gene, 2) understanding the gene content and evolutionary history of a newly discovered virus, or 3) identifying the organisms present in environments such as contaminated soils or the human gut.	○	○
8. Using a drawing, explain the structure and function of the CRISPR/Cas9 system and its application to human gene editing.	○	○
9. Given a gene sequence, design a CRISPR/Cas9 system that could be used to disable the gene.	○	○
10. Evaluate ethical and safety concerns associated with genetically modified organisms, including human gene therapy.	○	○
11. Evaluate the ethical issues involved in obtaining and using data on DNA sequences and chromosome structure in human parents and fetuses.	○	○

Please share any feedback you have about the content or wording of the above learning objectives.

Demographic Questions

Do you have Biology Education Research experience?

Yes No

Other (Please Specify)

To what extent have the Vision and Change **Core Concepts** changed your Introductory Biology course design since the report was issued by the AAAS in 2011?

A Great Deal Some

Very Little None

To what extent have the Vision and Change **Core Competencies** changed your Introductory Biology for Majors course design since the report was issued by the AAAS in 2011?

A Great Deal Some

Very Little None

I most closely identify as

Woman Man

○ ○

Non-binary/Trans

○

Other (Please Specify)

☐

○

Prefer not to say

○

I most closely identify as

American Indian or Alaska Native

☐

Asian or Asian American

☐

Black or African American

☐

Hispanic, Latinx, or Spanish origin

☐

Native Hawaiian or Pacific Islander

☐

White

☐

Other (Please Specify)

☐

Prefer not to say

☐

Thank you for reviewing a block of assigned learning objectives for an Introductory Biology course for Majors. If you are interested in providing feedback on additional blocks of learning objectives, please select **REVIEW ADDITIONAL LOs.**

REVIEW ADDITIONAL LOs

○

FINISH SURVEY

○

If you have any questions about the project or this survey, please do not hesitate to contact me, Dr. Kelly Hennessey (hennek@uw.edu), or Dr. Scott Freeman (srf991@uw.edu).

Powered by Qualtrics

UNIT: EVOLUTION

We ask that you complete the following demographic questions so that we can determine if we are gathering feedback from a representative population. We will not link your responses with any individual identifying information when sharing the results of this survey.

Please skip to the bottom of this section if you are returning to the survey and have already provided us with this information.

What is the name of your current institution?

Which of the following best describes your institution type?

- Associate's Degree-Granting
- Bachelor's Degree-Granting
- Master's Degree-Granting
- Doctoral Degree-Granting
- Other (Please Specify)

Which of the following best describes your current position?

- Postdoc
- Lecturer, instructor, or teaching faculty
- Assistant, Associate, or Full Professor
- Staff

- ○ Other (Please Specify)

In your current position, what is your primary responsibility?

- ○ Teaching
- ○ Research
- ○ Teaching and Research Equally
- ○ Other (Please Describe Briefly)

What is or was the focus of your graduate training? (Please select all that apply.)

- ☐ Molecular/Cellular/Developmental Biology
- ☐ Anatomy/Physiology
- ☐ Ecology/Evolutionary Biology
- ☐ Discipline-Based Education Research
- ☐ Other (Please Specify)

In an average academic year when you are teaching, which levels do you teach? (Please select all that apply.)

- ☐ Non-Majors Lower-Level (100 - 200 Level)
- ☐ Majors Lower-Level (100 - 200 Level)
- ☐ Upper-Level (300 - 400 Level)
- ☐ Graduate-Level (500+ Level)

What is the primary focus of the <u>majority</u> of biology courses that you have taught? (Please select all that apply.)

- ☐ Biochemistry
- ☐ Cell Biology
- ☐ Genetics
- ☐ Evolution
- ☐ Biodiversity of Life
- ☐ Plant and Animal Physiology
- ☐ Ecology
- ☐ Other (Please Specify)

In a typical academic term *when you are teaching in an Introductory Biology for Majors series*, what is the <u>focus of the course you teach</u>? (Please select all that apply.)

- ☐ Biochemistry
- ☐ Cell Biology
- ☐ Genetics
- ☐ Evolution
- ☐ Biodiversity of Life
- ☐ Plant and Animal Physiology
- ☐ Ecology
- ☐ Other (Please Specify)

To what extent do you communicate learning objectives to your students in your introductory biology course? (Please select all that apply.)

☐ Every class session
☐ Weekly
☐ Unit overview
☐ Course overview
☐ Syllabus
☐ Other (Please Briefly Describe)

Evolution - TOPICS: Natural Selection; Other Processes

This block of ***twenty*** learning objectives is part of the **EVOLUTION** unit, under the topics:

- **NATURAL SELECTION**
- **OTHER EVOLUTIONARY PROCESSES**

Please note that the learning objectives you are evaluating are a subset of all of the learning objectives proposed for the unit.

Please indicate whether the following learning objectives are **essential** or **non-essential** for the course you teach.

TOPIC: NATURAL SELECTION

	ESSENTIAL	NON-ESSENTIAL
1. Define adaptation, fitness, evolution, and theory. For each term, explain how its use in science differs from its use in everyday English.	○	○

	ESSENTIAL	NON-ESSENTIAL
2. Explain why the fossil record and genetic and structural homologies provide evidence for evolution.	○	○
3. Explain the connection between mutation and heritable variation in traits, and how selection on this variation can lead to changes in allele frequencies.	○	○
4. Analyze data or design an experiment in the lab, greenhouse, or field on evolution by natural selection. Identify the model organism, treatment, control conditions, and outcome variable measured. Interpret the outcomes, or graph the predicted outcomes, for both treatments over many generations.	○	○
5. Define directional and stabilizing selection and explain how each influences the mean value of a trait and the variation around the mean.	○	○
6. Given data on how allele frequencies or trait means change over time in a population, identify episodes of directional and stabilizing selection.	○	○
7. Using specific examples, explain why evolution by natural selection is neither random nor progressive, and why adaptations are not 'perfect'.	○	○
8. Identify which types of traits sexual selection acts on and explain why sexual selection is considered a special case of natural selection.	○	○

	ESSENTIAL	NON-ESSENTIAL
9. Explain why in some species, females are larger and more colorful than males.	○	○

Please share any feedback you have about the content or wording of the above learning objectives.

Please indicate whether the following learning objectives are **essential** or **non-essential** for the course you teach.

TOPIC: OTHER EVOLUTIONARY PROCESSES

	ESSENTIAL	NON-ESSENTIAL
1. Define genetic drift and describe how it influences allele frequencies. Explain why it is more important in small populations than in large populations and why it eventually leads to fixation or loss of alleles. Provide examples of events or processes that cause drift.	○	○
2. Analyze a scenario or design an experiment in a laboratory setting where drift is more important than natural selection as an evolutionary process.	○	○
3. Define gene flow and describe how it impacts allele frequencies in the source and recipient population.	○	○

	ESSENTIAL	NON-ESSENTIAL
4. Analyze a scenario or design an experiment in a laboratory setting where gene flow is more important than natural selection as an evolutionary process.	○	○
5. Defend the statement "mutation is the ultimate source of genetic variation," and explain why mutation is random with respect to its impact on an individual's fitness.	○	○
6. Explain why mutation is a particularly important evolutionary process in bacteria, viruses, and other groups with short generation times.	○	○
7. Define non-random mating and explain why it changes genotype frequencies but not allele frequencies.	○	○
8. Define inbreeding. Explain why inbreeding depression occurs in some populations.	○	○
9. For a gene with a given number of alleles, identify the genotype frequencies expected under the Hardy-Weinberg principle.	○	○
10. Explain 1) why the Hardy-Weinberg principle provides a null model for evolution, and 2) why natural selection, genetic drift, gene flow, mutation, and/or non-random mating can each produce genotype frequencies different from those expected under the Hardy-Weinberg principle.	○	○

	ESSENTIAL	NON-ESSENTIAL
11. Given data on observed genotype frequencies, calculate the genotype frequencies expected under the Hardy-Weinberg conditions and interpret the difference or lack of difference between observed and expected values.	○	○

Please share any feedback you have about the content or wording of the above learning objectives.

Evolution - TOPICS: Tree Thinking; Speciation; History of Life; Synthesis

This block of ***twenty*** learning objectives is part of the **EVOLUTION** unit, under the topics:

- **TREE THINKING**
- **SPECIATION**
- **HISTORY OF LIFE**
- **SYNTHESIS**

Please note that the learning objectives you are evaluating are a subset of all of the learning objectives proposed for the unit.

Please indicate whether the following learning objectives are **essential** or **non-essential** for the course you teach.

TOPIC: TREE THINKING

	ESSENTIAL	NON-ESSENTIAL
1. Given a phylogenetic tree, 1) label the root, nodes, branches, and tips; 2) identify the type of taxon at the tips (e.g., species or larger group), 3) circle and label several monophyletic groups, at least two of which are nested, 4) state at least one synapomorphy that identifies each of the circled monophyletic groups, and 5) find the most recent common ancestor of any two given taxa.	○	○
2. Given a phylogenetic tree and information on the state of a trait like flower shape or leg shape at each tip--including one or more outgroups--create a hypothesis for how that trait changed over time. Your analysis should include identifying possible instances of homoplasy.	○	○
3. Explain how parsimony 1) is used as a criterion in logic, 2) relates to the concepts of homology and homoplasy, and 3) can be used to estimate phylogenetic trees.	○	○
4. Given a dataset showing the presence or absence of morphological or molecular traits for a small group of taxa, estimate the tree most consistent with the data.	○	○

Please share any feedback you have about the content or wording of the above learning objectives.

Please indicate whether the following learning objectives are **essential** or **non-essential** for the course you teach.

TOPIC: SPECIATION

	ESSENTIAL	NON-ESSENTIAL
1. Compare the strengths and limitations of the biological, morphological, and phylogenetic species concepts.	○	○
2. Given the relevant data, determine whether identified populations are of the same or different species under the biological, morphological, and phylogenetic species concepts.	○	○
3. Explain 1) why genetic isolation and genetic divergence lead to speciation, and 2) the roles that gene flow, natural selection, genetic drift, and mutation can play in speciation.	○	○
4. Analyze data or design an experiment on how two populations may be evolving into distinct species.	○	○

Please share any feedback you have about the content or wording of the above learning objectives.

Please indicate whether the following learning objectives are **essential** or **non-essential** for the course you teach.

TOPIC: HISTORY OF LIFE

	ESSENTIAL	NON-ESSENTIAL
1. Define mass extinction and give examples.	○	○

	ESSENTIAL	NON-ESSENTIAL
2. Evaluate data on competing hypotheses for the causes of the end-Cretaceous and/or end-Permian mass extinctions.	○	○
3. Given data on the rate of recent species losses, do calculations or other analyses to evaluate the claim that a mass extinction event is currently underway. Be sure to evaluate sources of 1) bias in the data you are working with, and 2) uncertainty in your estimate.	○	○
4. Using a map of the world, diagram the central claims of the Out of Africa hypothesis. As you do so, explain the role of genetic drift, natural selection, and gene flow in the evolution of Homo sapiens.	○	○
5. Evaluate data on the genetic similarities and differences among existing human populations, in light of the claim that races named on the basis of traits like skin or hair color or facial features are "real" in terms of the underlying biology.	○	○
6. Define adaptive radiation and use examples to explain how ecological opportunities and/or key innovations can cause them.	○	○
7. Based on the information provided, evaluate the claim that a given group represents adaptive radiation.	○	○

Please share any feedback you have about the content or wording of the above learning objectives.

Please indicate whether the following learning objectives are **essential** or **non-essential** for the course you teach.

TOPIC: SYNTHESIS

	ESSENTIAL	NON-ESSENTIAL
1. Explain how gene flow, inbreeding, and genetic drift may positively or negatively affect endangered species that live in isolated (fragmented) habitats.	○	○
2. Given data on a specific endangered species, evaluate the advantages and disadvantages of creating gene flow by moving individuals into a population that is declining and communicate a recommendation to a stakeholder group.	○	○
3. Define fitness trade-off. Using drug resistance, herbicide resistance, or another example, explain why the evolution of a trait is likely to impose a fitness trade-off.	○	○
4. Using your knowledge or provided information, propose a strategy for slowing the evolution of drug resistance, herbicide resistance, or another trait that impacts human health and welfare.	○	○
5. Given a phylogeny and information about a trait in species included, mark the tree to indicate where changes in the trait occurred.	○	○

Please share any feedback you have about the content or wording of the above learning objectives.

Demographic Questions

Do you have Biology Education Research experience?

Yes No

Other (Please Specify)

To what extent have the Vision and Change **Core Concepts** changed your Introductory Biology course design since the report was issued by the AAAS in 2011?

A Great Deal Some

Very Little None

To what extent have the Vision and Change **Core Competencies** changed your Introductory Biology for Majors course design since the report was issued by the AAAS in 2011?

A Great Deal Some

Very Little None

I most closely identify as

Woman Man

○ ○

Non-binary/Trans

○

Other (Please Specify)

○

Prefer not to say

○

I most closely identify as

American Indian or Alaska Native

☐

Asian or Asian American

☐

Black or African American

☐

Hispanic, Latinx, or Spanish origin

☐

Native Hawaiian or Pacific Islander

☐

White

☐

Other (Please Specify)

☐

Prefer not to say

☐

Thank you for reviewing a block of assigned learning objectives for an Introductory Biology course for Majors. If you are interested in providing feedback on additional blocks of learning objectives, please select **REVIEW ADDITIONAL LOs.**

REVIEW ADDITIONAL LOs

○

FINISH SURVEY

○

If you have any questions about the project or this survey, please do not hesitate to contact me, Dr. Kelly Hennessey (hennek@uw.edu), or Dr. Scott Freeman (srf991@uw.edu).

Powered by Qualtrics

Respondent Characteristics

UNIT: BIODIVERSITY OF LIFE

We ask that you complete the following demographic questions so that we can determine if we are gathering feedback from a representative population. We will not link your responses with any individual identifying information when sharing the results of this survey.

Please skip to the bottom of this section if you are returning to the survey and have already provided us with this information.

What is the name of your current institution?

Which of the following best describes your institution type?

- Associate's Degree-Granting
- Bachelor's Degree-Granting
- Master's Degree-Granting
- Doctoral Degree-Granting
- Other (Please Specify)

Which of the following best describes your current position?

- Postdoc
- Lecturer, instructor, or teaching faculty
- Assistant, Associate, or Full Professor
- Staff

- ○ Other (Please Specify)

In your current position, what is your primary responsibility?

- ○ Teaching
- ○ Research
- ○ Teaching and Research Equally
- ○ Other (Please Describe Briefly)

What is or was the focus of your graduate training? (Please select all that apply.)

- ☐ Molecular/Cellular/Developmental Biology
- ☐ Anatomy/Physiology
- ☐ Ecology/Evolutionary Biology
- ☐ Discipline-Based Education Research
- ☐ Other (Please Specify)

In an average academic year when you are teaching, which levels do you teach? (Please select all that apply.)

- ☐ Non-Majors Lower-Level (100 - 200 Level)
- ☐ Majors Lower-Level (100 - 200 Level)
- ☐ Upper-Level (300 - 400 Level)
- ☐ Graduate-Level (500+ Level)

What is the primary focus of the majority of biology courses that you have taught? (Please select all that apply.)

- ☐ Biochemistry
- ☐ Cell Biology
- ☐ Genetics
- ☐ Evolution
- ☐ Biodiversity of Life
- ☐ Plant and Animal Physiology
- ☐ Ecology
- ☐ Other (Please Specify)

In a typical academic term *when you are teaching in an Introductory Biology for Majors series*, what is the focus of the course you teach? (Please select all that apply.)

- ☐ Biochemistry
- ☐ Cell Biology
- ☐ Genetics
- ☐ Evolution
- ☐ Biodiversity of Life
- ☐ Plant and Animal Physiology
- ☐ Ecology
- ☐ Other (Please Specify)

To what extent do you communicate learning objectives to your students in your introductory biology course? (Please select all that apply.)

- [ ] Every class session
- [ ] Weekly
- [ ] Unit overview
- [ ] Course overview
- [ ] Syllabus
- [ ] Other (Please Briefly Describe)

Biodiversity of Life - Tree of Life; Bacteria/Archaea; Fungi; Viruses

This block of ***twenty-three*** learning objectives is part of the **BIODIVERSITY OF LIFE** unit, under the topics:

- **TREE OF LIFE**
- **BACTERIA AND ARCHAEA**
- **FUNGI**
- **VIRUSES**

Please note that the learning objectives you are evaluating are a subset of all of the learning objectives proposed for the unit.

Please indicate whether the following learning objectives are **essential** or **non-essential** for the course you teach.

TOPIC: TREE OF LIFE

	ESSENTIAL	NON-ESSENTIAL
1. Construct a tree showing the relationships among the three domains and label it with synapomorphies.	○	○

	ESSENTIAL	NON-ESSENTIAL
2. Classify an organism that you've never heard of before, by domain, when given a set of its characteristics. Explain your reasoning.	○	○
3. Explain the major differences between bacteria and fungi (or similar pair of familiar organisms) to a 10-year-old.	○	○
4. Given a set of traits, identify a species that you've never encountered before as a virus, "protist," plant, fungus, or animal, and explain your reasoning.	○	○

Please share any feedback you have about the content or wording of the above learning objectives.

Please indicate whether the following learning objectives are **essential** or **non-essential** for the course you teach.

TOPIC: BACTERIA AND ARCHAEA

	ESSENTIAL	NON-ESSENTIAL
1. Discuss examples of structural and functional adaptations in bacteria and archaea that enable these organisms to exploit a wide array of energy sources and thrive in many different environments.	○	○

	ESSENTIAL	NON-ESSENTIAL
2. Given a set of environmental conditions, predict at least two adaptations that allow bacteria and archaea to live in that habitat.	○	○
3. Explain each of the major steps in an experiment designed to identify the bacteria and archaea present in a sample from an environment of interest such as a cow's gut, the deep sea, or a hot spring.	○	○
4. Analyze and interpret a data set on the bacteria and archaea present in samples taken before and after an environmental change, such as the same human gut before and after antibiotic use, or the same soil before and after contamination with crude oil.	○	○
5. Describe at least three of the beneficial and harmful roles that bacteria and archaea play in the lives of humans and the biosphere as a whole.	○	○
6. Explain why antibiotics specifically kill bacteria instead of the host, and why antibiotics that kill many different species of bacteria tend to cause side effects for the host.	○	○

Please share any feedback you have about the content or wording of the above learning objectives.

Please indicate whether the following learning objectives are **essential** or **non-essential** for the course you teach.

TOPIC: FUNGI

	ESSENTIAL	NON-ESSENTIAL
1. On a tree showing the major lineages of fungi, label where the following synapomorphies arose: fruiting bodies that are cup-like, arbuscular associations with plant root cells, ectomycorrhizal associations with plant root cells, cell walls with chitin, and fruiting bodies that include mushrooms.	○	○
2. Analyze the adaptations that make some fungi efficient parasites, and explain why fungal diseases in animals are difficult to treat.	○	○
3. Using a drawing or other graphic, explain why fungal hyphae and mycelia have extremely high surface-area-to-volume ratios.	○	○
4. Create a flowchart, concept map, or another type of graphic that explains the connections among the following ideas: high surface-area-to-volume ratio, absorptive lifestyle, extracellular digestion, efficient decomposers, efficient parasites, mycorrhizal associations.	○	○
5. Use a drawing or other graphic to explain the mutually beneficial relationships between fungi and plants.	○	○
6. Analyze data or design an experiment on variation in the quantity of nutrients that fungi deliver to host plants.	○	○

Please share any feedback you have about the content or wording of the above learning objectives.

Please indicate whether the following learning objectives are **essential** or **non-essential** for the course you teach.

TOPIC: VIRUSES

	ESSENTIAL	NON-ESSENTIAL
1. Explain why viruses are considered obligate intracellular parasites, and why viral diseases are difficult to treat with drugs.	O	O
2. State the arguments for why viruses could be considered alive or not alive, then state and defend your opinion on this question.	O	O
3. Compare enveloped and nonenveloped viruses and give examples of viral genes that are not composed of DNA.	O	O
4. Describe the central dogma using the terms DNA, mRNA, proteins, transcription, translation, and explain at least two ways that viruses can contradict the central dogma.	O	O
5. Using a diagram, explain how the following events in a virus' life cycle occur: enter a host cell, produce viral proteins, replicate viral genome, assemble new virions, exit host cell, transmission to a new host.	O	O
6. Propose methods to disrupt each of the following events in a virus' life cycle: enter a host cell, produce viral proteins, replicate viral genome, assemble new virions, exit host cell, transmission to a new host. Each method should avoid harm to the host.	O	O

	ESSENTIAL	NON-ESSENTIAL
7. Explain why close physical contact is required for a virus to "jump" from one host species to another and predict the nature of mutations that enable a virus to parasitize cells in the new host.	○	○

Please share any feedback you have about the content or wording of the above learning objectives.

Biodiversity of Life - Eukaryotic Radiation ("Protists"); Plants; Animals

This block of ***seventeen*** learning objectives is part of the **BIODIVERSITY OF LIFE** unit, under the topics:

- **EUKARYOTIC RADIATION (PROTISTS)**
- **PLANTS**
- **ANIMALS**

Please note that the learning objectives you are evaluating are a subset of all of the learning objectives proposed for the unit.

Please indicate whether the following learning objectives are **essential** or **non-essential** for the course you teach.

TOPIC: EUKARYOTIC RADIATION (PROTISTS)

	ESSENTIAL	NON-ESSENTIAL
1. Describe the key characteristics of the common ancestor of all eukaryotes living today.	○	○
2. Using information provided on the traits of several major lineages, construct or label a tree of the eukaryotic radiation to show where key synapomorphies arose.	○	○
3. Explain the process of endosymbiosis and using your knowledge or information provided on a specific example, describe the costs and benefits to each organism involved.	○	○
4. Compare endosymbiosis with examples of horizontal gene transfer among bacteria.	○	○
5. Using information that you gather or are provided, describe the unique structural and functional characteristics of a specific protist species, and explain its medical or ecological importance.	○	○
6. Given maps of current and predicted global temperatures and the current distribution of a eukaryote of interest, predict changes in its geographic range and discuss potential consequences.	○	○

Please share any feedback you have about the content or wording of the above learning objectives.

Please indicate whether the following learning objectives are **essential** or **non-essential** for the course you teach.

TOPIC: PLANTS

	ESSENTIAL	NON-ESSENTIAL
1. Explain why the following sources of evidence support the hypothesis that land plants evolved from freshwater green algae: 1) morphological data, 2) the fossil record, and 3) the phylogenetic tree.	○	○
2. On a tree showing the relationships among major lineages of green algae and land plants, circle the most species-rich tip and label where the following synapomorphies arose: vascular tissue, flowers, stomata, cuticle, seeds, cellulose cell walls, pores, chloroplasts. Explain the adaptive significance of each synapomorphy.	○	○
3. Summarize the major features of alternation of generations.	○	○
4. Generate a hypothesis to explain the adaptive significance of alternation of generations (which is not well understood!)	○	○
5. Describe how plants influence the environment in terms of 1) atmospheric composition, 2) food availability in terrestrial environments, 3) soil formation, and 4) the quality and abundance of water in soils, marshes, and streams.	○	○

	ESSENTIAL	NON-ESSENTIAL
6. Predict the impact of extensive deforestation or other changes in plant communities in terms of 1) atmospheric composition, 2) food availability in terrestrial environments, 3) soil formation, and/or 4) the quality and abundance of water in soils, marshes, and streams.	○	○

Please share any feedback you have about the content or wording of the above learning objectives.

Please indicate whether the following learning objectives are **essential** or **non-essential** for the course you teach.

TOPIC: ANIMALS

	ESSENTIAL	NON-ESSENTIAL
1. On a tree that shows the major lineages of animals, label where the following synapomorphies arose: bilateral symmetry, cephalization, multicellularity, movement via contractile proteins. Explain each synapomorphy's adaptive significance. Identify the two most species-rich lineages.	○	○
2. Using information that you gather or are provided, characterize the following in a particular major animal lineage: 1) body plan, 2) reproductive systems, 3) sensory organs, 4) feeding strategies, and 5) ecological role.	○	○

	ESSENTIAL	NON-ESSENTIAL
3. On a tree showing the major lineages of chordates, label where the following synapomorphies arose: lungs, limbs, bone, notochord, muscular, post-anal tail, dorsal hollow nerve chord, fur, scales, cranium, and vertebrae. Explain each synapomorphy's adaptive significance.	○	○
4. Explain the benefits and challenges of aquatic versus terrestrial living in terms of gas exchange, movement, availability of food (for animals) or light (for plants), and keeping cells moist.	○	○
5. Generate a hypothesis to explain why terrestrial living evolved many times in animals versus once in sessile (non-moving) photosynthetic organisms such as plants.	○	○

Please share any feedback you have about the content or wording of the above learning objectives.

Demographic Questions

Do you have Biology Education Research experience?

Yes ○ No ○

Other (Please Specify) ○

To what extent have the Vision and Change **Core Concepts** changed your Introductory Biology course design since the report was issued by the AAAS in 2011?

A Great Deal ○ Some ○

Very Little ○ None ○

To what extent have the Vision and Change **Core Competencies** changed your Introductory Biology for Majors course design since the report was issued by the AAAS in 2011?

A Great Deal ○ Some ○

Very Little ○ None ○

I most closely identify as

Woman ○ Man ○

Non-binary/Trans ○

Other (Please specify) [ ] ○

Prefer not to say ○

I most closely identify as

American Indian or Alaska Native

☐

Asian or Asian American

☐

Black or African American

☐

Hispanic, Latinx, or Spanish origin

☐

Native Hawaiian or Pacific Islander

☐

White

☐

Other (Please Specify)

☐

Prefer not to say

☐

Thank you for reviewing a block of assigned learning objectives for an Introductory Biology course for Majors. If you are interested in providing feedback on additional blocks of learning objectives, please select **REVIEW ADDITIONAL LOs.**

REVIEW ADDITIONAL LOs

○

FINISH SURVEY

○

If you have any questions about the project or this survey, please do not hesitate to contact me, Dr. Kelly Hennessey (hennek@uw.edu), or Dr. Scott Freeman (srf991@uw.edu).

Powered by Qualtrics

Respondent Characteristics

UNIT: ECOLOGY

We ask that you complete the following demographic questions so that we can determine if we are gathering feedback from a representative population. We will not link your responses with any individual identifying information when sharing the results of this survey.

Please skip to the bottom of this section <u>if you are returning to the survey and have already provided us with this information</u>.

What is the name of your current institution?

[ ]

Which of the following <u>best</u> describes your institution type?

- ○ Associate's Degree-Granting
- ○ Bachelor's Degree-Granting
- ○ Master's Degree-Granting
- ○ Doctoral Degree-Granting
- ○ Other (Please Specify)

[ ]

Which of the following best describes your <u>current</u> position?

- ○ Postdoc
- ○ Lecturer, instructor, or teaching faculty
- ○ Assistant, Associate, or Full Professor
- ○ Staff

- ○ Other (Please Specify)

In your current position, what is your primary responsibility?

- ○ Teaching
- ○ Research
- ○ Teaching and Research Equally
- ○ Other (Please Describe Briefly)

What is or was the focus of your graduate training? (Please select all that apply.)

- ☐ Molecular/Cellular/Developmental Biology
- ☐ Anatomy/Physiology
- ☐ Ecology/Evolutionary Biology
- ☐ Discipline-Based Education Research
- ☐ Other (Please Specify)

In an average academic year when you are teaching, which levels do you teach? (Please select all that apply.)

- ☐ Non-Majors Lower-Level (100 - 200 Level)
- ☐ Majors Lower-Level (100 - 200 Level)
- ☐ Upper-Level (300 - 400 Level)
- ☐ Graduate-Level (500+ Level)

What is the primary focus of the majority of biology courses that you have taught? (Please select all that apply.)

- ☐ Biochemistry
- ☐ Cell Biology
- ☐ Genetics
- ☐ Evolution
- ☐ Biodiversity of Life
- ☐ Plant and Animal Physiology
- ☐ Ecology
- ☐ Other

In a typical academic term *when you are teaching in an Introductory Biology for Majors series*, what is the focus of the course you teach? (Please select all that apply.)

- ☐ Biochemistry
- ☐ Cell Biology
- ☐ Genetics
- ☐ Evolution
- ☐ Biodiversity of Life
- ☐ Plant and Animal Physiology
- ☐ Ecology
- ☐ Other (Please Specify)

To what extent do you communicate learning objectives to your students in your introductory biology course? (Please select all that apply.)

☐ Every class session
☐ Weekly
☐ Unit overview
☐ Course overview
☐ Syllabus
☐ Other (Please Briefly Describe)

Ecology: Introduction; Matter/Energy; Biodiversity/Conserv Ecology

This block of ***twenty-four*** learning objectives is part of the **ECOLOGY** unit, under the topics:

- **INTRODUCTION**
- **MATTER AND ENERGY IN ECOSYSTEMS (NUTRIENT CYCLING, PRODUCTIVITY)**
- **BIODIVERSITY & CONSERVATION BIOLOGY**

Please note that the learning objectives you are evaluating are a subset of all of the learning objectives proposed for the unit.

Please indicate whether the following learning objectives are **essential** or **non-essential** for the course you teach.

TOPIC: INTRODUCTION

	ESSENTIAL	NON-ESSENTIAL
1. Explain the difference between abiotic and biotic factors in an ecosystem and provide examples of how these factors can affect the communities present.	○	○

	ESSENTIAL	NON-ESSENTIAL
2. Given information on a particular biome, identify the most important biotic and abiotic factors present and explain why they rank as most important.	○	○
3. Compare the most important biotic and abiotic factors that limit the distribution and abundance of aquatic species versus terrestrial species.	○	○
4. Given predictions for how climate change will impact the planet over the next 50 years, predict how the distribution and abundance of aquatic species and terrestrial species will change in response.	○	○
5. Create a diagram explaining the nested relationships among populations, species, communities, and ecosystems.	○	○

Please share any feedback you have about the content or wording of the above learning objectives.

Please indicate whether the following learning objectives are **essential** or **non-essential** for the course you teach.

TOPIC: MATTER AND ENERGY IN ECOSYSTEMS (NUTRIENT CYCLING, PRODUCTIVITY)

	ESSENTIAL	NON-ESSENTIAL
1. Describe the major events in the global cycling of water, carbon, and nitrogen.	○	○
2. Explain how human activities have affected global nutrient cycles.	○	○
3. Analyze data or design an experiment on the consequences of adding or removing a specific nutrient in an ecosystem.	○	○
4. Compare net primary productivity in aquatic and terrestrial ecosystems.	○	○
5. Explain why there is typically less biomass and fewer individuals and species at the top of a food chain than at the bottom.	○	○
6. Predict the consequences of changes in primary production due to perturbations such as drought, fire, flood, extreme temperatures, or nutrient influx or loss.	○	○
7. Create a diagram showing the difference between top-down and bottom-up regulation in a community.	○	○
8. Describe how biomagnification is related to both trophic levels and food webs.	○	○

	ESSENTIAL	NON-ESSENTIAL
9. Create a strategy designed to mitigate the negative effects of biomagnification in a particular species or community.	○	○
10. Explain the logic behind the claim that eating at the primary producer level is better for the environment than eating at higher trophic levels.	○	○

Please share any feedback you have about the content or wording of the above learning objectives.

Please indicate whether the following learning objectives are **essential** or **non-essential** for the course you teach.

TOPIC: BIODIVERSITY & CONSERVATION BIOLOGY

	ESSENTIAL	NON-ESSENTIAL
1. Explain how biologists measure biodiversity, how species richness differs from species diversity, and why plant diversity is often used as an indicator of overall biodiversity.	○	○
2. Analyze data or design a study on current biodiversity at a site and changes in biodiversity over time.	○	○

	ESSENTIAL	NON-ESSENTIAL
3. Describe the latitudinal gradient in biodiversity and explain the criteria that researchers used to identify a biodiversity hotspot.	○	○
4. Create a presentation, skit, essay, or video segment explaining how people who live far from biodiverse regions can work with local people and governments to preserve biodiversity, especially when there are large disparities in wealth in the two groups.	○	○
5. Using predictions from theory and data, provide three reasons why diversity influences productivity.	○	○
6. Create a presentation, skit, essay, or video segment to 4th graders that explain the most compelling reasons to preserve biodiversity and prevent a 6th mass extinction.	○	○
7. Explain how habitat loss, habitat fragmentation, climate change, over-harvesting, and invasive species affect biodiversity.	○	○
8. Evaluate or propose a plan to preserve biodiversity in a given area.	○	○
9. Give an example of an indicator species and describe how it serves as a measure of the environmental conditions where it lives.	○	○

Please share any feedback you have about the content or wording of the above learning objectives.

Ecology: Populations; Species; Climate Change

This block of ***twenty-four*** learning objectives is part of the **ECOLOGY** unit, under the topics:

- **POPULATIONS**
- **SPECIES INTERACTIONS**
- **CLIMATE CHANGE**

Please note that the learning objectives you are evaluating are a subset of all of the learning objectives proposed for the unit.

Please indicate whether the following learning objectives are **essential** or **non-essential** for the course you teach.

TOPIC: POPULATIONS

	ESSENTIAL	NON-ESSENTIAL
1. Given information presented as graphs, tables, or equations, describe how the size of a population has changed over time.	○	○
2. Given information on a species' history and current status, use mathematical tools to predict changes in its population size over time.	○	○

	ESSENTIAL	NON-ESSENTIAL
3. Use diagrams to explain the difference between clumped, uniform, and random distributions of individuals within a species, and state which type of distribution is most common.	○	○
4. Analyze the consequences of human activities changing a species' distribution from the continuous occupation of a broad area to occupying small, disconnected patches within the same area.	○	○

Please share any feedback you have about the content or wording of the above learning objectives.

Please indicate whether the following learning objectives are **essential** or **non-essential** for the course you teach.

TOPIC: SPECIES INTERACTIONS & COMMUNITY ECOLOGY

	ESSENTIAL	NON-ESSENTIAL
1. Explain why life-history traits, competition, predation, parasitism, mutualism, and niche breadth (abiotic factors) impact the distribution and abundance of a given species.	○	○

	ESSENTIAL	NON-ESSENTIAL
2. Given information on changes in intraspecific competition, interspecific competition, resource abundance, predation, parasitism, or mutualism, predict how a specific population will evolve and/or acclimate in response.	○	○
3. Explain why life-history traits, competition, predation, parasitism, mutualism, and niche breadth (abiotic factors) impact which species are found in the same community.	○	○
4. Analyze the consequences of human activities changing a community's distribution from the continuous occupation of a broad area to occupying small, disconnected patches within the same area.	○	○
5. Using specific examples, explain why biotic factors create differences between fundamental and realized niches.	○	○
6. Analyze data or design an experiment on the hypothesis that a specified biotic factor creates a difference between a species' fundamental and realized niche.	○	○
7. Give an example of how the composition of a community could change based on its geographic size (spatial scale) and explain why this might occur.	○	○

	ESSENTIAL	NON-ESSENTIAL
8. Analyze data or design a study on how changing spatial or temporal scale changes our understanding of the composition and dynamics of a specific community.	○	○
9. Give an example of how the composition of a community has changed over time and describe evidence or hypotheses that explain why this change occurred.	○	○
10. Given a food web or other information on relationships among species in that community, predict the consequences of removing an apex ("top") predator, introducing a specific disease, or other specified changes occurring.	○	○
11. Explain why fitness trade-offs lead to dramatically different life-history traits in early successional (pioneering or "weedy") versus late-successional (climax) species.	○	○
12. Predict the results of changing the frequency or intensity of disturbance in a community, given information on species composition and species traits.	○	○

Please share any feedback you have about the content or wording of the above learning objectives.

Please indicate whether the following learning objectives are **essential** or **non-essential** for the course you teach.

TOPIC: CLIMATE CHANGE

	ESSENTIAL	NON-ESSENTIAL
1. Explain how human activities impact climate change by disrupting the global carbon cycle.	○	○
2. Using a diagram of the global carbon cycle, propose a practical, evidence-based, and multi-pronged strategy for decreasing CO_2 emissions into the atmosphere and increasing deposition of CO_2 into "sinks."	○	○
3. Describe the role of feedback loops in climate change and provide examples of both positive and negative feedback for a given greenhouse gas.	○	○
4. Interpret graphs that show changes in atmospheric CO_2 over the past 200 years and the past 10 years.	○	○
5. Given predictions of future changes in carbon emissions, evaluate the impacts for organisms and ecosystems.	○	○
6. Explain how climate warming leads to changes in the timing of seasonal events (phenology) and how changes in the phenology of one species can affect the ecology of other species.	○	○

	ESSENTIAL	NON-ESSENTIAL
7. Use data to evaluate whether a species of interest will be impacted by changes in its phenology--either directly or via changes that occur in species that it interacts with.	○	○
8. Explain the logic behind the acronym MAD (move, acclimate/adapt, die) that summarizes how species are responding to climate change.	○	○

Please share any feedback you have about the content or wording of the above learning objectives.

Demographic Questions

Do you have Biology Education Research experience?

Yes	No
○	○

Other (Please Specify) ○

To what extent have the Vision and Change **Core Concepts** changed your Introductory Biology course design since the report was issued by the AAAS in 2011?

A Great Deal Some

○ ○

Very Little None

○ ○

To what extent have the Vision and Change **Core Competencies** changed your Introductory Biology for Majors course design since the report was issued by the AAAS in 2011?

A Great Deal Some

○ ○

Very Little None

○ ○

I most closely identify as

Woman Man

○ ○

Non-binary/Trans

○

Other (Please Specify)

[ ]

○

Prefer not to say

○

I most closely identify as

American Indian or Alaska Native

☐

Asian or Asian American

☐

Black or African American

☐

Hispanic, Latinx, or Spanish origin

☐

Native Hawaiian or Pacific Islander

☐

White

☐

Other (Please Specify)

☐

Prefer not to say

☐

Thank you for reviewing a block of assigned learning objectives for an Introductory Biology course for Majors. If you are interested in providing feedback on additional blocks of learning objectives, please select **REVIEW ADDITIONAL LOs.**

REVIEW ADDITIONAL LOs

○

FINISH SURVEY

○

If you have any questions about the project or this survey, please do not hesitate to contact me, Dr. Kelly Hennessey (hennek@uw.edu), or Dr. Scott Freeman (srf991@uw.edu).

Powered by Qualtrics

UNIT: PLANT AND ANIMAL PHYSIOLOGY

We ask that you complete the following demographic questions so that we can determine if we are gathering feedback from a representative population. We will not link your responses with any individual identifying information when sharing the results of this survey.

Please skip to the bottom of this section if you are returning to the survey and have already provided us with this information.

What is the name of your current institution?

Which of the following best describes your institution type?

- Associate's Degree-Granting
- Bachelor's Degree-Granting
- Master's Degree-Granting
- Doctoral Degree-Granting
- Other (Please Specify)

Which of the following best describes your current position?

- Postdoc
- Lecturer, instructor, or teaching faculty
- Assistant, Associate, or Full Professor
- Staff

○ Other (Please Specify)

In your current position, what is your primary responsibility?

○ Teaching
○ Research
○ Teaching and Research Equally
○ Other (Please Describe Briefly)

What is or was the focus of your graduate training? (Please select all that apply.)

☐ Molecular/Cellular/Developmental Biology
☐ Anatomy/Physiology
☐ Ecology/Evolutionary Biology
☐ Discipline-Based Education Research
☐ Other (Please Specify)

In an average academic year when you are teaching, which levels do you teach? (Please select all that apply.)

☐ Non-Majors Lower-Level (100 - 200 Level)
☐ Majors Lower-Level (100 - 200 Level)
☐ Upper-Level (300 - 400 Level)
☐ Graduate-Level (500+ Level)

What is the primary focus of the majority of biology courses that you have taught? (Please select all that apply.)

- ☐ Biochemistry
- ☐ Cell Biology
- ☐ Genetics
- ☐ Evolution
- ☐ Biodiversity of Life
- ☐ Plant and Animal Physiology
- ☐ Ecology
- ☐ Other (Please Specify)

In a typical academic term *when you are teaching in an Introductory Biology for Majors series*, what is the focus of the course you teach? (Please select all that apply.)

- ☐ Biochemistry
- ☐ Cell Biology
- ☐ Genetics
- ☐ Evolution
- ☐ Biodiversity of Life
- ☐ Plant and Animal Physiology
- ☐ Ecology
- ☐ Other (Please Specify)

To what extent do you communicate learning objectives to your students in your introductory biology course? (Please select all that apply.)

- [ ] Every class session
- [ ] Weekly
- [ ] Unit overview
- [ ] Course overview
- [ ] Syllabus
- [ ] Other (Please Briefly Describe)

Animal-Specific Physiology - Overview; Cell Membrane; Electrical Signal; Homeos

This block of ***twenty*** learning objectives is part of the **PLANT AND ANIMAL PHYSIOLOGY** unit, under the topics:

- **OVERVIEW**
- **ANIMAL-SPECIFIC PHYSIOLOGY - CELL MEMBRANE**
- **ANIMAL-SPECIFIC PHYSIOLOGY - ELECTRICAL SIGNALING**
- **ANIMAL-SPECIFIC PHYSIOLOGY - HOMEOSTASIS**

Please note that the learning objectives you are evaluating are a subset of all of the learning objectives proposed for the unit.

Please indicate whether the following learning objectives are **essential** or **non-essential** for the course you teach.

TOPIC: OVERVIEW

	ESSENTIAL	NON-ESSENTIAL
1. Describe how a given cell type combines with others to form a tissue with emergent properties, how that tissue and other tissues combine to form an organ with emergent properties, and how that organ and other organs combine to form a physiological system with emergent properties.	○	○
2. Analyze the relationships among the cells, tissues, and/or organs involved in a given physiological system, including how their structures correlate with their functions and how they interact in terms of function.	○	○
3. Using a specific cell, tissue, organ, or system, explain the relationship between surface area and volume in heat transfer, gas exchange, nutrient transport, or some other physiological function in terms that a 4th grader can understand.	○	○
4. Use graphs and/or equations to demonstrate the relationship between surface area and volume, then relate the axes on the graph and/or parameters in the equation to elements in a specific animal cell, tissue, organ, or system.	○	○
5. Describe how the structures and functions of a given cell type, tissue type, organ, or physiological system have been modified, via evolution by natural selection, in ways that allow species to occupy different habitats or acquire resources in a different way.	○	○

	ESSENTIAL	NON-ESSENTIAL
6. Analyze the fitness trade-offs and/or historical (evolutionary) constraints on a given cell type, tissue, organ, or physiological system that prevents it from fulfilling its function more effectively or efficiently.	○	○

Please share any feedback you have about the content or wording of the above learning objectives.

Please indicate whether the following learning objectives are **essential** or **non-essential** for the course you teach.

TOPIC: ANIMAL-SPECIFIC PHYSIOLOGY: CELL MEMBRANE

	ESSENTIAL	NON-ESSENTIAL
1. Explain the basic structure and function of epithelial tissue, including the nature of polarity in epithelial cells and the role of tight junctions between cells.	○	○
2. Create a model of an epithelial cell, given information on its function and a list of the membrane channels, pumps, and carriers available in that organism.	○	○
3. Using diagrams, explain the difference between voltage-gated ion channels and ligand-dated ion channels.	○	○

	ESSENTIAL	NON-ESSENTIAL
4. Given a specific structural change, predict how the function of a voltage-gated channel or ligand-gated channel would change.	○	○

Please share any feedback you have about the content or wording of the above learning objectives.

Please indicate whether the following learning objectives are **essential** or **non-essential** for the course you teach.

TOPIC: ANIMAL-SPECIFIC PHYSIOLOGY: ELECTRICAL SIGNALING

	ESSENTIAL	NON-ESSENTIAL
1. Draw a generalized version of a neuron. Label the dendrites, cell body, an axon and describe the function of each.	○	○
2. Explain 1) what it means to say that an action potential is an all-or-none event, and 2) how specific information can be represented by the frequency and source of action potentials received by a cell.	○	○
3. Given data on the action potentials received by a cell predict whether the cell will fire an action potential in response.	○	○

	ESSENTIAL	NON-ESSENTIAL
4. Explain how changes in membrane potential that occur during an action potential depend on the activity of voltage-gated channels, and how an action potential propagates along a cell.	○	○
5. Given information on a specific neurotoxin, predict how it will impact the frequency of action potentials.	○	○
6. Explain how the information in an action potential is transduced at a synapse and then transmitted to a post-synaptic cell.	○	○
7. Given information on a specific neurotoxin, predict how it will impact the transmission of action potentials.	○	○
8. Compare chemical and electrical signals in terms of 1) the speed of transmission, 2) location of transmission, and 3) how an individual signal exists in a network of other signals.	○	○

Please share any feedback you have about the content or wording of the above learning objectives.

Please indicate whether the following learning objectives are **essential** or **non-essential** for the course you teach.

TOPIC: ANIMAL-SPECIFIC PHYSIOLOGY: HOMEOSTASIS

	ESSENTIAL	NON-ESSENTIAL
1. For a given homeostatically regulated variable, create a flow chart or other model showing the relationships among the stimulus, sensor, input message (afferent pathway), integrator (controller), output message (efferent pathway), target (effector), and response. Add notes on the function of each component and highlight examples of negative feedback in this system.	○	○
2. For a given homeostatic system, predict how a given change in one or more of its components will change the activity of the system.	○	○

Please share any feedback you have about the content or wording of the above learning objectives.

Plant and Animal Physiology - Flow Down Grad; Cell/Cell Comm; Mass Bal; Homeos

This block of ***twenty-seven*** learning objectives is part of the **PLANT AND ANIMAL PHYSIOLOGY** unit, under the topics:

- **FLOW DOWN GRADIENTS**
- **CELL TO CELL COMMUNICATION**
- **MASS BALANCE**
- **HOMEOSTASIS**

Please note that the learning objectives you are evaluating are a subset of all of the learning objectives proposed for the unit.

Please indicate whether the following learning objectives are **essential** or **non-essential** for the course you teach.

TOPIC: FLOW DOWN GRADIENTS

	ESSENTIAL	NON-ESSENTIAL
1. Explain how and why ions and molecules move in response to concentration gradients.	○	○
2. In a given system, identify the mechanism responsible for the movement of matter or heat and predict the direction, path, and relative rate of movement.	○	○
3. Explain how ions move in response to electrical potential gradients, and why.	○	○
4. Based on the information you are given about a specific cell, predict how it will be affected by ion movements that occur in response to a change in electrical potential gradients.	○	○
5. Explain how fluids and gases move in response to pressure gradients, and why.	○	○
6. Given information about a particular system, predict the physiological responses to specified changes in pressure gradients.	○	○

	ESSENTIAL	NON-ESSENTIAL
7. Explain how heat moves in response to temperature gradients, and why.	○	○
8. Given data on temperature gradients in a given physiological system, predict the movement of heat.	○	○
9. Define resistance and give an example at the cellular level and an example at the systems or whole-organism level.	○	○
10. In a given system, identify the source(s) of resistance, explain why it impedes movement, and analyze one or more mechanisms that reduce or increase resistance.	○	○
11. Explain how and why flux depends on the size of a gradient and the magnitude of resistance and give an example from a specific physiological system.	○	○
12. Predict how given changes in gradients and/or resistance will change flux in a system.	○	○

Please share any feedback you have about the content or wording of the above learning objectives.

Please indicate whether the following learning objectives are **essential** or **non-essential** for the course you teach.

TOPIC: CELL TO CELL COMMUNICATION

	ESSENTIAL	NON-ESSENTIAL
1. Compare the structure and solubility of peptide, steroid, and amine messengers, then compare how they are transported in the blood and enter cells.	○	○
2. Explain the relationship between a chemical messenger and its receptor, and why only certain cells respond to a specific messenger.	○	○
3. Predict how a given change in a receptor will alter a cell's response.	○	○
4. Make a simple diagram illustrating the signal transduction, signal amplification, and response events that occur after a messenger arrives at or in a cell and include the role of second messengers if relevant.	○	○
5. Predict how a given change in a signal transduction pathway will alter the cellular response.	○	○
6. Explain why different cell types may respond differently to the same messenger molecule.	○	○
7. Explain why chemical messengers can elicit a large response even though they are present at very low concentrations.	○	○

	ESSENTIAL	NON-ESSENTIAL
8. Create analogies or other approaches to communicate the concepts that chemical messages are graded (not all-or-none), that responses to messengers are graded and time-limited, that the same messenger can elicit more than one type of response from the same cell, and that cells integrate information from many chemical messengers--often at the same time.	○	○
9. Compare the speed and duration of responses from messengers that act via second messengers to messengers that act via changes in gene expression.	○	○
10. Describe how the signal from a chemical messenger ends.	○	○
11. Given information about a specific messenger system, predict the consequences of a specific change in signal termination.	○	○

Please share any feedback you have about the content or wording of the above learning objectives.

Please indicate whether the following learning objectives are **essential** or **non-essential** for the course you teach.

TOPIC: MASS BALANCE

	ESSENTIAL	NON-ESSENTIAL
1. Give an example of a compartment in an animal or plant body that contains a volume of physiologically important gas, liquid, or solute, and describe how matter enters and leaves the compartment.	○	○
2. Given information on flow rate and concentration or partial pressure, use equations to calculate the mass entering or leaving a compartment.	○	○

Please share any feedback you have about the content or wording of the above learning objectives.

Please indicate whether the following learning objectives are **essential** or **non-essential** for the course you teach.

TOPIC: HOMEOSTASIS

	ESSENTIAL	NON-ESSENTIAL
1. For a given homeostatically regulated property, explain how positive and negative feedback maintains it at a normal range and why that is important to the organism's fitness.	○	○
2. For a given homeostatic system, state a plausible, scientific (testable) hypothesis to explain why a different organism does not regulate this variable.	○	○

	ESSENTIAL	NON-ESSENTIAL

Please share any feedback you have about the content or wording of the above learning objectives.

Plant-Specific - Structure/Function; Sensing/Respond; Growth/Repro; Matter/Ener

This block of ***twenty-one*** learning objectives is part of the **PLANT AND ANIMAL PHYSIOLOGY** unit, under the topics:

- **PLANT-SPECIFIC PHYSIOLOGY: STRUCTURE AND FUNCTION**
- **PLANT-SPECIFIC PHYSIOLOGY: SENSING AND RESPONDING TO ENVIRONMENTAL CHANGE**
- **PLANT-SPECIFIC PHYSIOLOGY: GROWTH AND REPRODUCTION**
- **PLANT-SPECIFIC PHYSIOLOGY: MATTER AND ENERGY FLOW**

Please note that the learning objectives you are evaluating are a subset of all of the learning objectives proposed for the unit.

Please indicate whether the following learning objectives are **essential** or **non-essential** for the course you teach.

TOPIC: PLANT-SPECIFIC PHYSIOLOGY: STRUCTURE AND FUNCTION

	ESSENTIAL	NON-ESSENTIAL
1. Explain the roles of cellulose, lignin, and other components of the cell wall matrix in support, growth, and protection against pathogens.	○	○

	ESSENTIAL	NON-ESSENTIAL
2. In a drawing or photograph of a plant you have never seen before, identify roots, stems, and leaves and explain their overall function.	○	○
3. Given drawings or photographs and information on the habitat occupied by a plant you've never seen before, suggest hypotheses for the adaptive (functional) significance of its root, stem, and leaf structures.	○	○

Please share any feedback you have about the content or wording of the above learning objectives.

Please indicate whether the following learning objectives are **essential** or **non-essential** for the course you teach.

TOPIC: PLANT-SPECIFIC PHYSIOLOGY: SENSING AND RESPONDING TO ENVIRONMENTAL CHANGE

	ESSENTIAL	NON-ESSENTIAL
1. Predict how the root, stem, and leaf structures in a plant will change in response to a given change in the environment.	○	○

	ESSENTIAL	NON-ESSENTIAL
2. In a given diagram showing a plant's reaction to an environmental stimulus (e.g., a change in gravity, the direction or quality of light, availability of water, or pathogen or herbivore activity), identify and explain the role of the sensor, signal transduction pathway(s), hormonal signal(s), and response(s).	○	○
3. Given information about the system in question, predict how a specified change in a sensor, signal transduction pathway, or hormonal signal will change how a plant responds to the environment.	○	○
4. Describe how stomata (paired guard cells) allow for CO_2 and O_2 exchange and explain how and why they change shape in response to changes in the environment.	○	○
5. Under given light, water, or temperature conditions, predict adaptations or developmental responses in stomata and other cells and structures that optimize the rate of photosynthesis and minimize the rate of water loss.	○	○
6. Explain the adaptive significance of sensing and responding to a molecule from another plant that is released after an attack by a predator or pathogen.	○	○

	ESSENTIAL	NON-ESSENTIAL
7. Analyze data or design an experiment to test the hypothesis that a given molecule acts as a signal in communication between different plants.	○	○

Please share any feedback you have about the content or wording of the above learning objectives.

Please indicate whether the following learning objectives are **essential** or **non-essential** for the course you teach.

TOPIC: PLANT-SPECIFIC PHYSIOLOGY: GROWTH & REPRODUCTION

	ESSENTIAL	NON-ESSENTIAL
1. Given a diagram of a familiar plant lifecycle, label when and where mitosis, meiosis, and fertilization occur, and identify haploid and diploid phases.	○	○
2. Given a diagram of the life cycle of a plant you have never seen before, label when and where meiosis and fertilization occur and identify haploid and diploid phases.	○	○
3. Compare primary and secondary growth, using diagrams to indicate the locations of meristematic tissues.	○	○

	ESSENTIAL	NON-ESSENTIAL
4. Define "hormone", then identify several plant hormones and explain their function at the cellular, tissue, organ, system, or whole-organism level.	○	○
5. Explain the role of auxin or other growth hormones in regulating the overall shape or size of a plant.	○	○

Please share any feedback you have about the content or wording of the above learning objectives.

Please indicate whether the following learning objectives are **essential** or **non-essential** for the course you teach.

TOPIC: PLANT-SPECIFIC PHYSIOLOGY: MATTER AND ENERGY FLOW

	ESSENTIAL	NON-ESSENTIAL
1. Create a diagram, drawing, or model to communicate how carbon is assimilated into organic compounds in plants and relate this to the flow of energy and the synthesis of molecules required for maintenance and growth.	○	○
2. Explain how the CO_2 in "weightless" air is the source of mass in a redwood tree to an 8-year-old.	○	○

	ESSENTIAL	NON-ESSENTIAL
3. Create a drawing or other model to explain how inorganic nutrients are obtained from soil, either directly or via associations with mycorrhizal fungi.	○	○
4. Compare the structure and function of the cells and tissues involved in sugar transport (phloem) versus water transport (xylem).	○	○
5. Compare the mechanisms responsible for the long-distance transport of sugars versus water and nutrients.	○	○
6. Predict how increased soil salt concentrations or other changes affect the movement of water in plants.	○	○

Please share any feedback you have about the content or wording of the above learning objectives.

Demographic Questions

Do you have Biology Education Research experience?

Yes No

Other (Please Specify)

To what extent have the Vision and Change **Core Concepts** changed your Introductory Biology course design since the report was issued by the AAAS in 2011?

A Great Deal Some

Very Little None

To what extent have the Vision and Change **Core Competencies** changed your Introductory Biology for Majors course design since the report was issued by the AAAS in 2011?

A Great Deal Some

Very Little None

I most closely identify as

Woman Man

Non-binary/Trans

Other (Please Specify)

[ ]

○

Prefer not to say

○

I most closely identify as

American Indian or Alaska Native

☐

Asian or Asian American

☐

Black or African American

☐

Hispanic, Latinx, or Spanish origin

☐

Native Hawaiian or Pacific Islander

☐

White

☐

Other (Please Specify)

☐

Prefer not to say

☐

Thank you for reviewing a block of assigned learning objectives for an Introductory Biology course for Majors. If you are interested in providing feedback on additional blocks of learning objectives, please select **REVIEW ADDITIONAL LOs.**

REVIEW ADDITIONAL LOs

○

FINISH SURVEY

○

If you have any questions about the project or this survey, please do not hesitate to contact me, Dr. Kelly Hennessey (hennek@uw.edu), or Dr. Scott Freeman (srf991@uw.edu).

Powered by Qualtrics
